# Supplementary figures and images for: Evaluating beta-tubulin variants as predictors of benzimidazole resistance across Caenorhabditis nematodes
Source: PLoS Pathog. 2026 Jun 5;22(6):e1014306. doi: 10.1371/journal.ppat.1014306 (PMC13262929; doi:10.1371/journal.ppat.1014306)

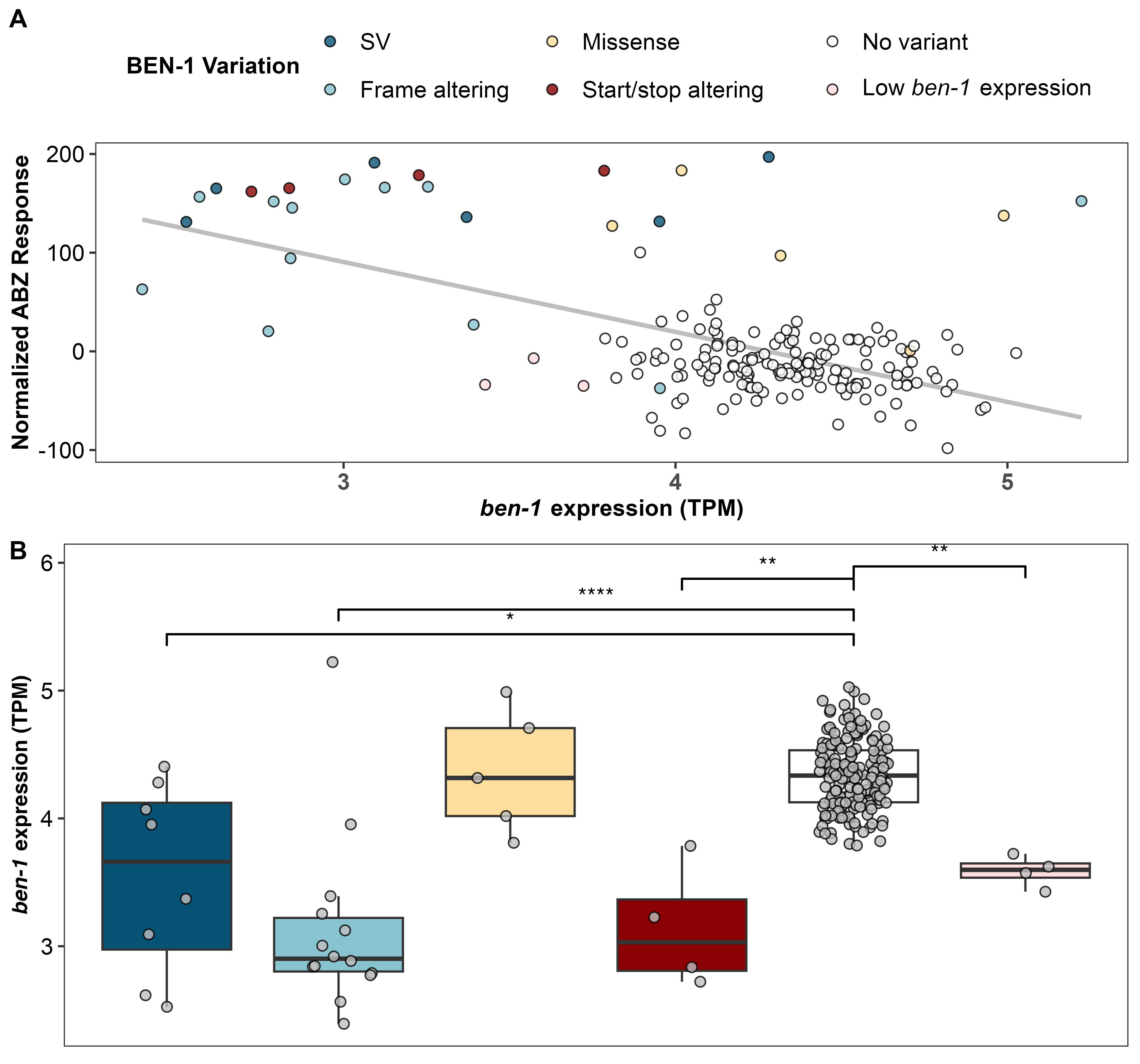

Supplement: S1 Fig — (A) Scatterplot of the relationship between ben-1 expression levels and normalized albendazole (ABZ) response across C. elegans wild strains. Each point represents a strain phenotyped for ABZ response in previous publications [14, 36] with ben-1 expression data [37]. The ben-1 expression level measured in transcripts per million (TPM) is displayed on the x-axis. The normalized ABZ response values adjusted for assay-specific effects are displayed on the y-axis. The gray line represents the linear regression fit between ben-1 expression and normalized response (R2 = 0.34, p-value = 5.16e-18), with the linear model’s coefficient of determination (R²). Data points are colored based on the predicted functional consequence of the ben-1 allele for each strain (i.e., large structural variant (SV), frameshift, missense substitution, disrupted start/stop sequence, no high-impact variant, or low ben-1 expression). (B) Boxplots of ben-1 expression levels among strains grouped by the predicted functional consequences of their ben-1 alleles. Each point represents the ben-1 expression level of an individual within each group. We tested for statistically significant differences in the expression between each consequence type and wild strains without a high-impact ben-1 allele with an unpaired Wilcoxon test. Significance levels are indicated by symbols: ‘*’ (p < 0.05), ‘**’ (p < 0.01), ‘***’ (p < 0.001), ‘****’ (p < 0.0001). (TIFF) [file ppat.1014306.s001.tiff]

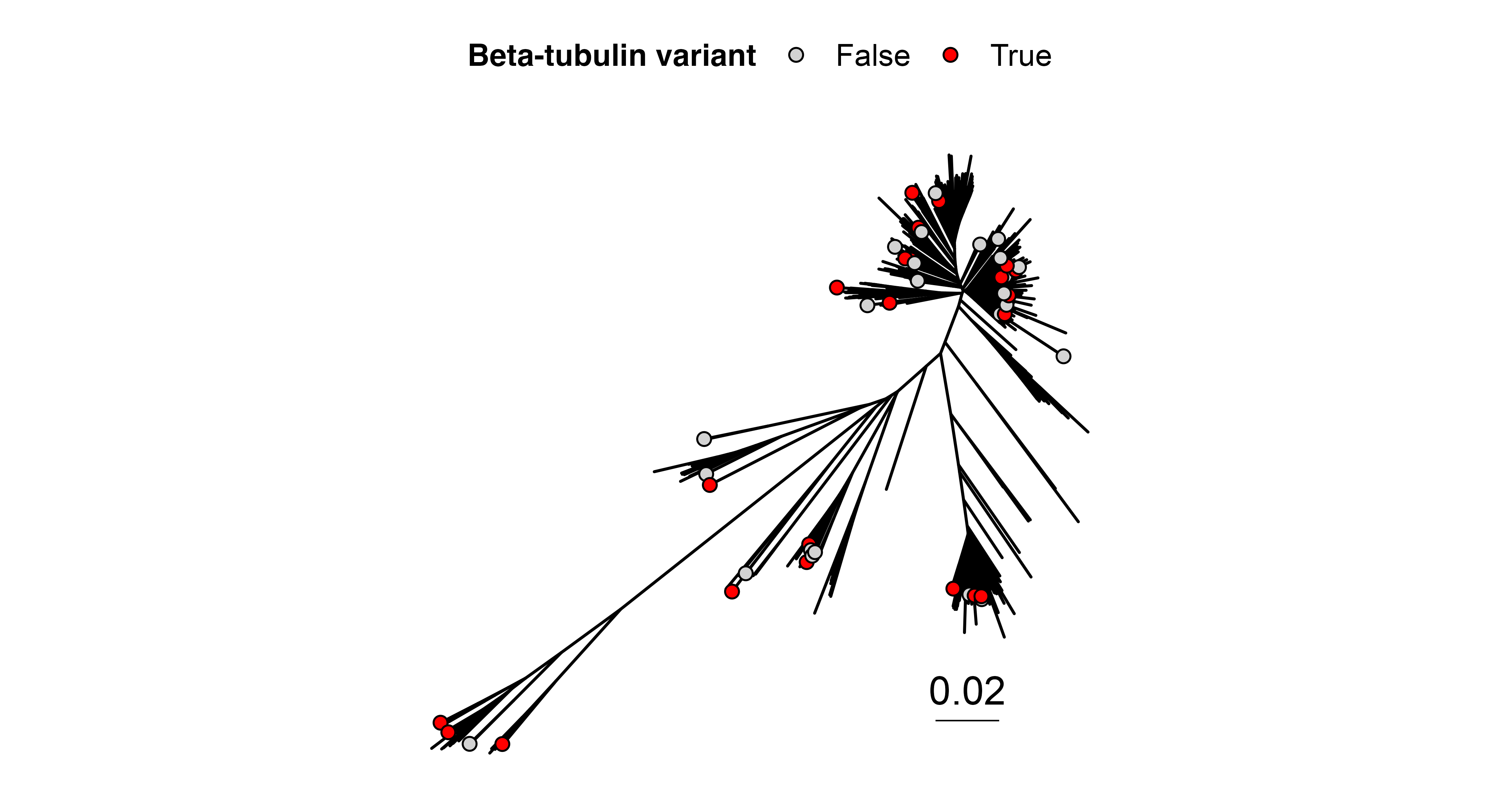

Supplement: S2 Fig — C. briggsae strains included in high-throughput larval development assays (HTLDAs) are highlighted on the C. briggsae species tree. Strains with predicted high-impact variants in a beta-tubulin gene are denoted by red points. Strains with no predicted variants in any beta-tubulin gene are denoted by gray points. (TIFF) [file ppat.1014306.s002.tiff]

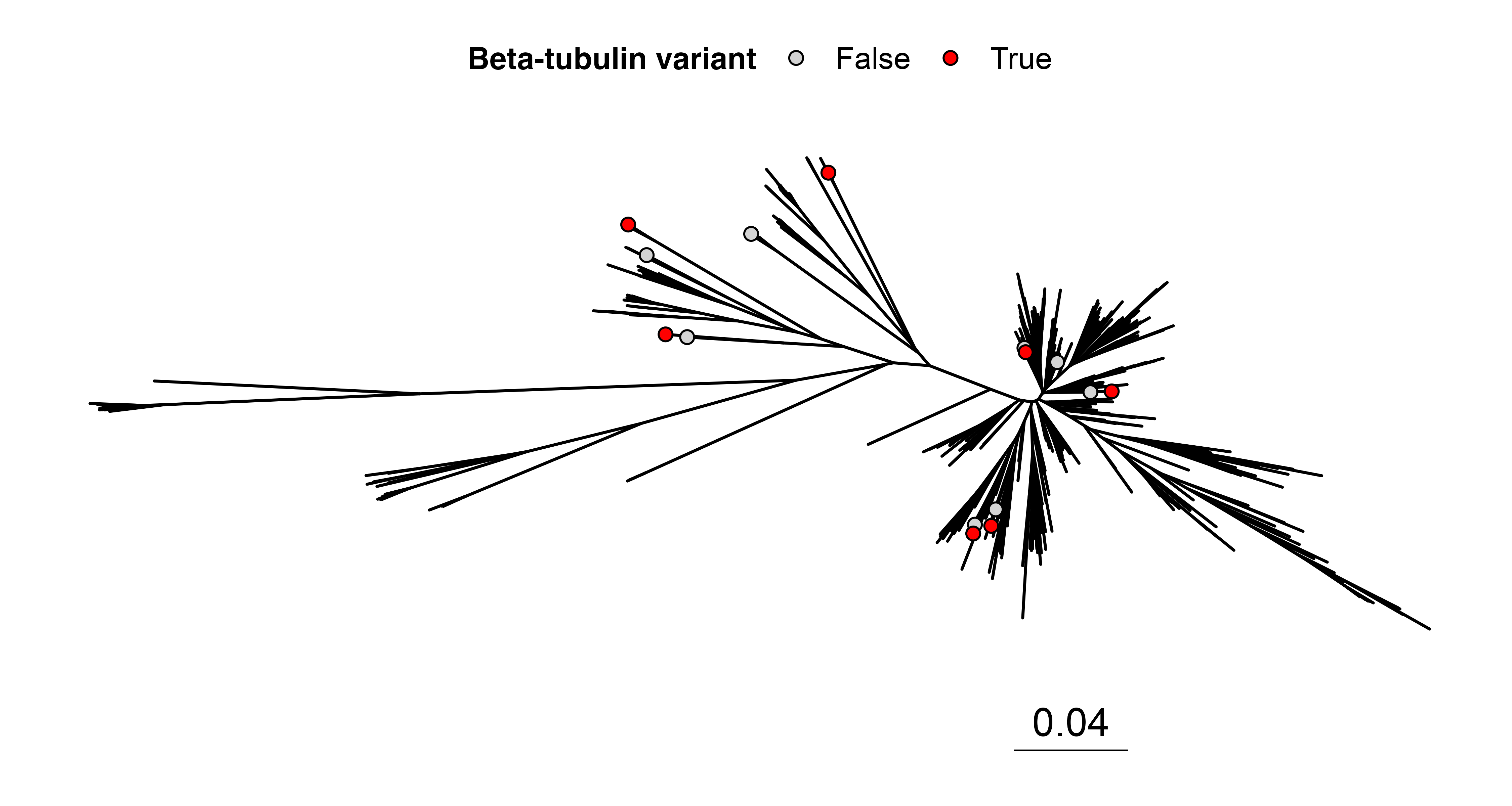

Supplement: S3 Fig — C. tropicalis strains included in high-throughput larval development assays (HTLDAs) are highlighted on the C. tropicalis species tree. Strains with predicted high-impact variants in a beta-tubulin gene are denoted by red points. Strains with no predicted variants in any beta-tubulin gene are denoted by gray points. (TIFF) [file ppat.1014306.s003.tiff]

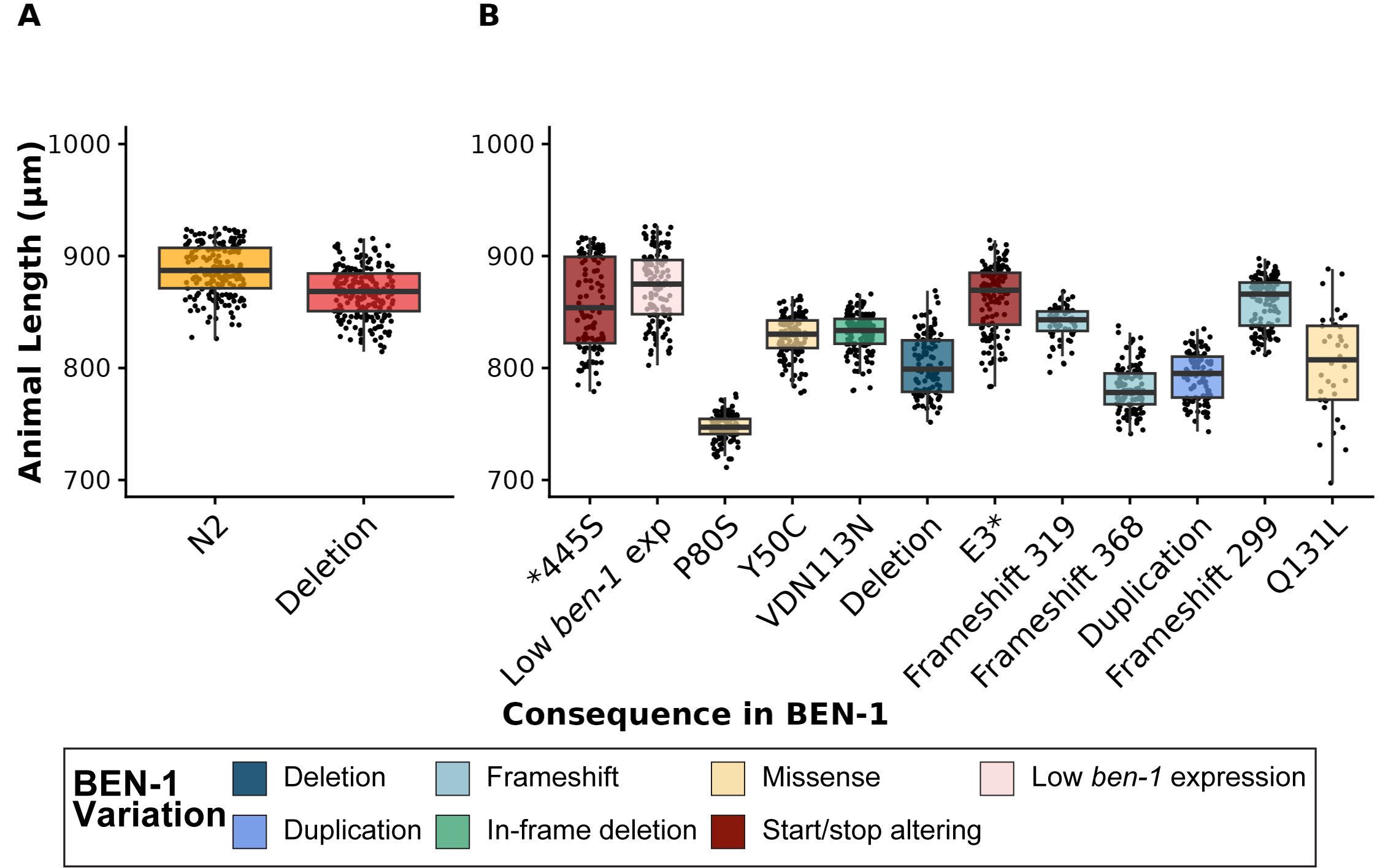

Supplement: S4 Fig — Median animal length values from populations of nematodes grown in DMSO are shown on the y-axis. Each point represents the median animal length from a well containing approximately five to 30 animals. Data are shown as Tukey box plots with the median as a solid horizontal line, the top and bottom of the box representing the 75th and 25th quartiles, respectively. The top whisker is extended to the maximum point that is within a 1.5 interquartile range from the 75th quartile. The bottom whisker is extended to the minimum point that is within the 1.5 interquartile range from the 25th quartile. Results for (A) the N2 reference strain (orange) and a strain with a ben-1 deletion in the N2 background (red), and (B) all wild C. elegans strains with unique high-impact variants in ben-1 are sorted by their relative resistance to ABZ based on median animal length. Wild C. elegans strains are colored by beta-tubulin variant status. (TIF) [file ppat.1014306.s004.tif]

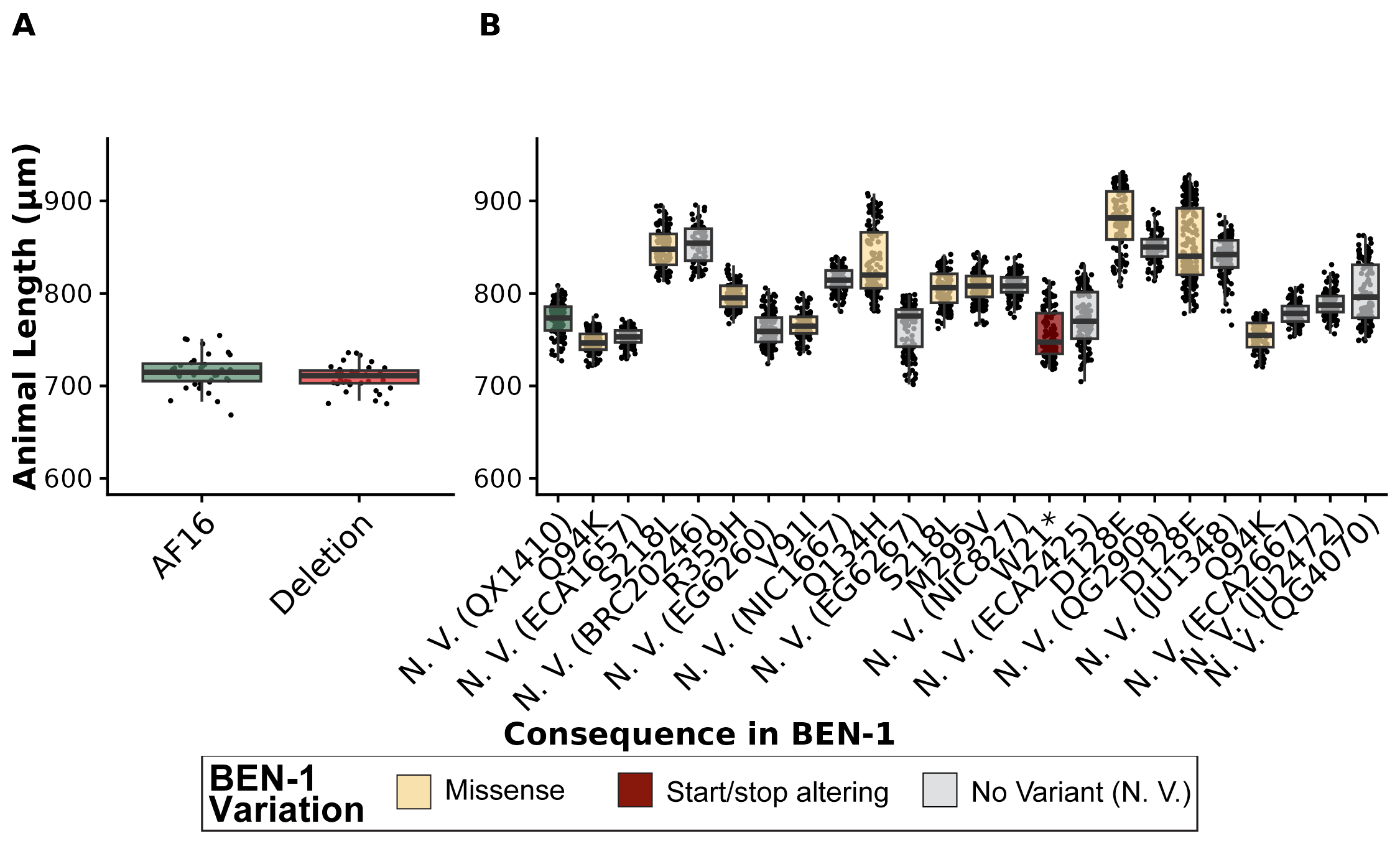

Supplement: S5 Fig — Median animal length values from populations of nematodes grown in DMSO are shown on the y-axis. Each point represents the median animal length from a well containing approximately five to 30 animals. Data are shown as Tukey box plots with the median as a solid horizontal line, the top and bottom of the box representing the 75th and 25th quartiles, respectively. The top whisker is extended to the maximum point that is within a 1.5 interquartile range from the 75th quartile. The bottom whisker is extended to the minimum point that is within the 1.5 interquartile range from the 25th quartile. Results for (A) the AF16 reference strain (green) and two strains each with an independent ben-1 deletion in the AF16 background (ECA3953 and ECA3954) (red), and (B) all wild C. briggsae strains with unique high-impact variants in ben-1 are sorted by their relative resistance to ABZ based on median animal length. No variant (N. V.) strains (gray) paired with strains that have a high-impact variant in a beta-tubulin gene are shown alongside each corresponding strain with a high-impact variant in a beta-tubulin gene. Wild C. briggsae strains are colored by beta-tubulin variant status. (TIF) [file ppat.1014306.s005.tif]

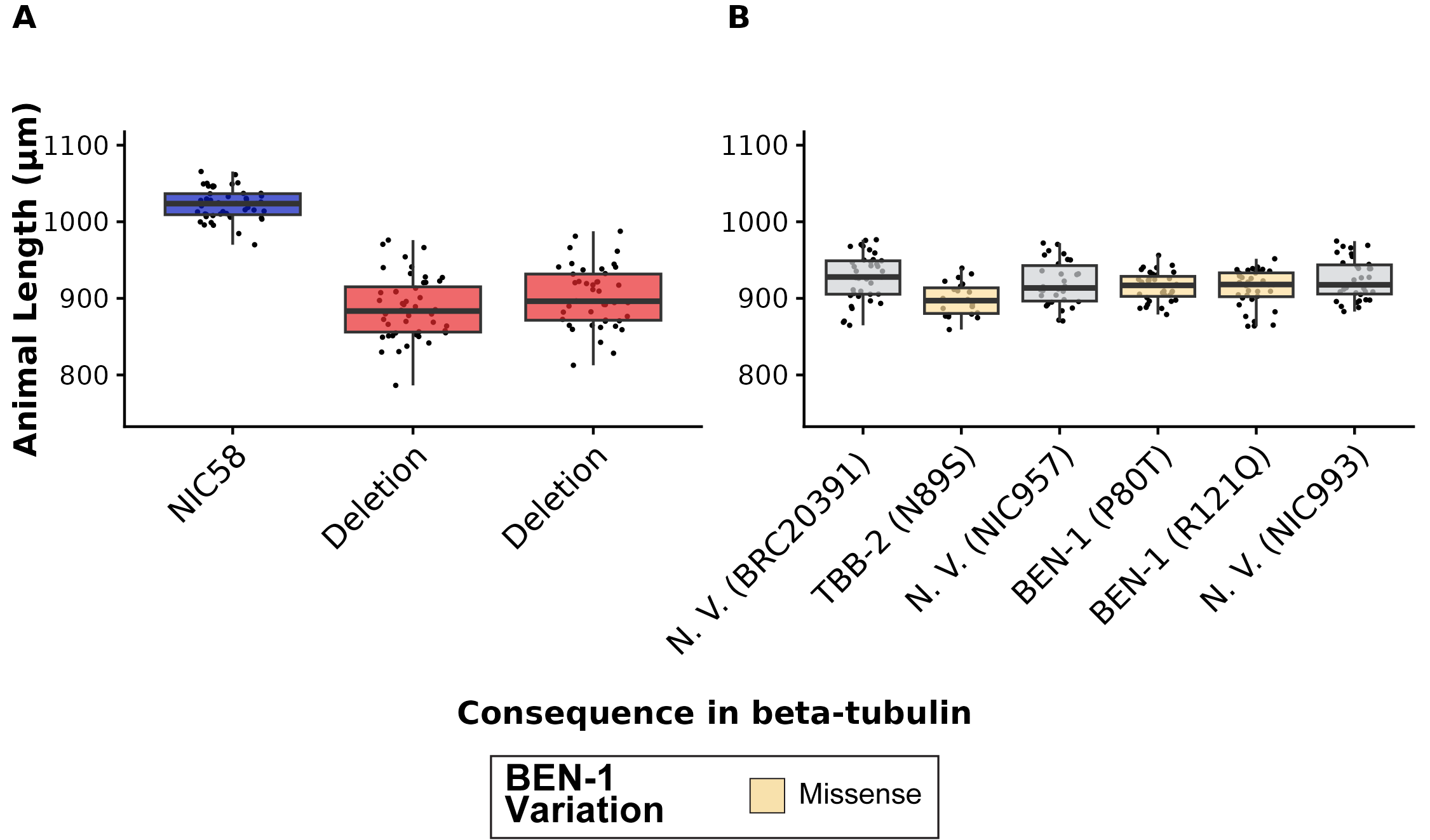

Supplement: S6 Fig — Median animal length values from populations of nematodes grown in DMSO are shown on the y-axis. Each point represents the median animal length from a well containing approximately five to 30 animals. Data are shown as Tukey box plots with the median as a solid horizontal line, the top and bottom of the box representing the 75th and 25th quartiles, respectively. The top whisker is extended to the maximum point that is within a 1.5 interquartile range from the 75th quartile. The bottom whisker is extended to the minimum point that is within the 1.5 interquartile range from the 25th quartile. Results for (A) the NIC58 reference strain (blue) and two strains each with an independent ben-1 deletion in the NIC58 background (ECA4247 and ECA4248) (red), and (B) all wild C. tropicalis strains with unique high-impact variants in ben-1 or tbb-2 are sorted by their relative resistance to ABZ based on median animal length. No variant (N. V.) strains (gray) paired with strains that have a high-impact variant in a beta-tubulin gene are shown alongside each corresponding strain with a high-impact variant in a beta-tubulin gene. Wild C. tropicalis strains are colored by beta-tubulin variant status. (TIF) [file ppat.1014306.s006.tif]

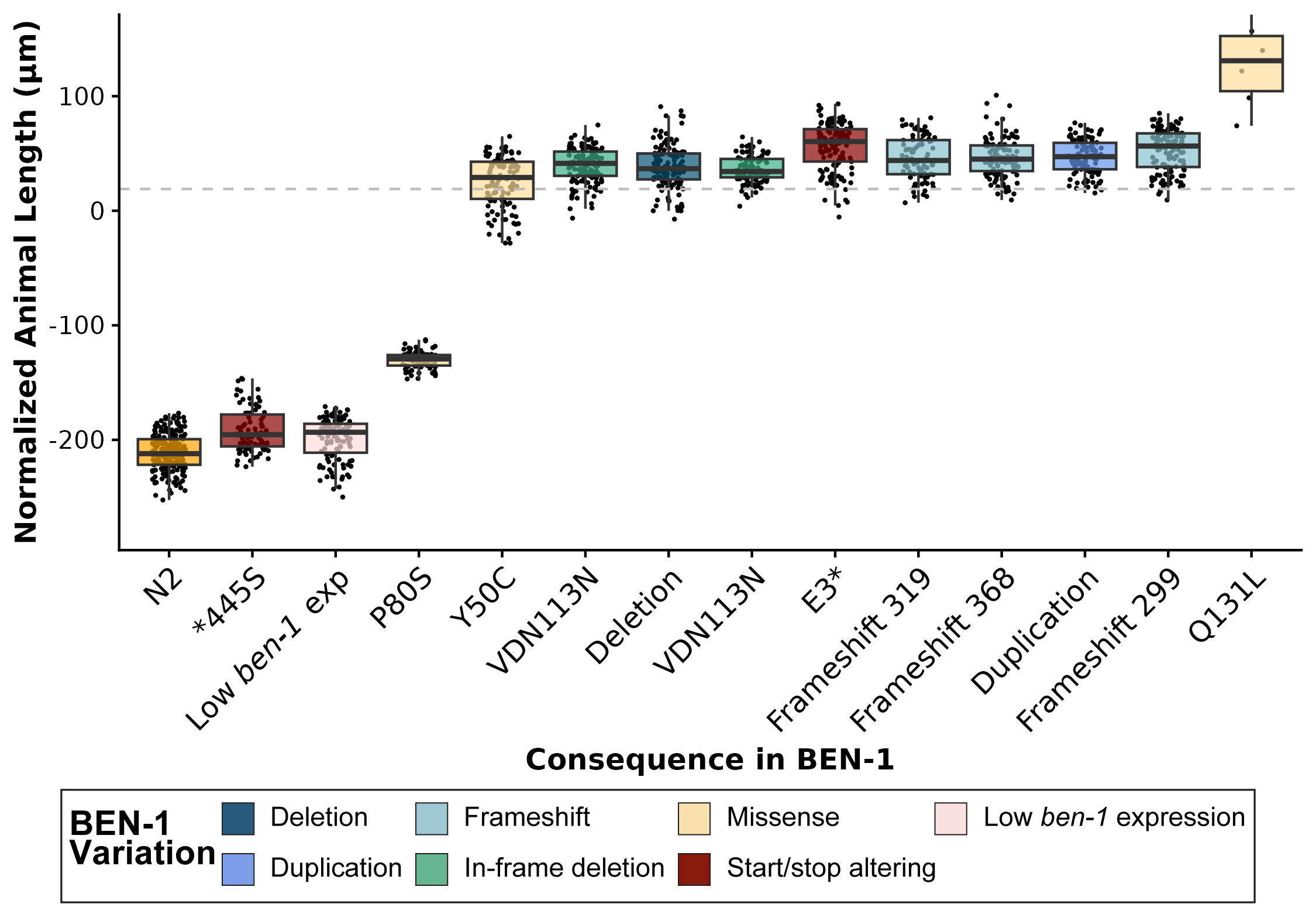

Supplement: S7 Fig — The regressed median animal length values for populations of nematodes grown in 30 μM albendazole (ABZ) are shown on the y-axis. Each point represents the normalized median animal length value of a well containing approximately five to 30 animals. Data are shown as Tukey box plots with the median as a solid horizontal line, and the top and bottom of the box representing the 75th and 25th quartiles, respectively. The top whisker is extended to the maximum point that is within the 1.5 interquartile range from the 75th quartile. The bottom whisker is extended to the minimum point that is within the 1.5 interquartile range from the 25th quartile. The gray dashed line marks the C. elegans resistance threshold, defined as two standard deviations below the mean of the ben-1 deletion strain in the N2 reference strain background. Results for the N2 reference strain (orange) and all wild C. elegans strains with unique high-impact variants in ben-1 are sorted by their relative resistance to ABZ based on median animal length. Wild C. elegans strains are colored by beta-tubulin variant status. (TIF) [file ppat.1014306.s007.tif]

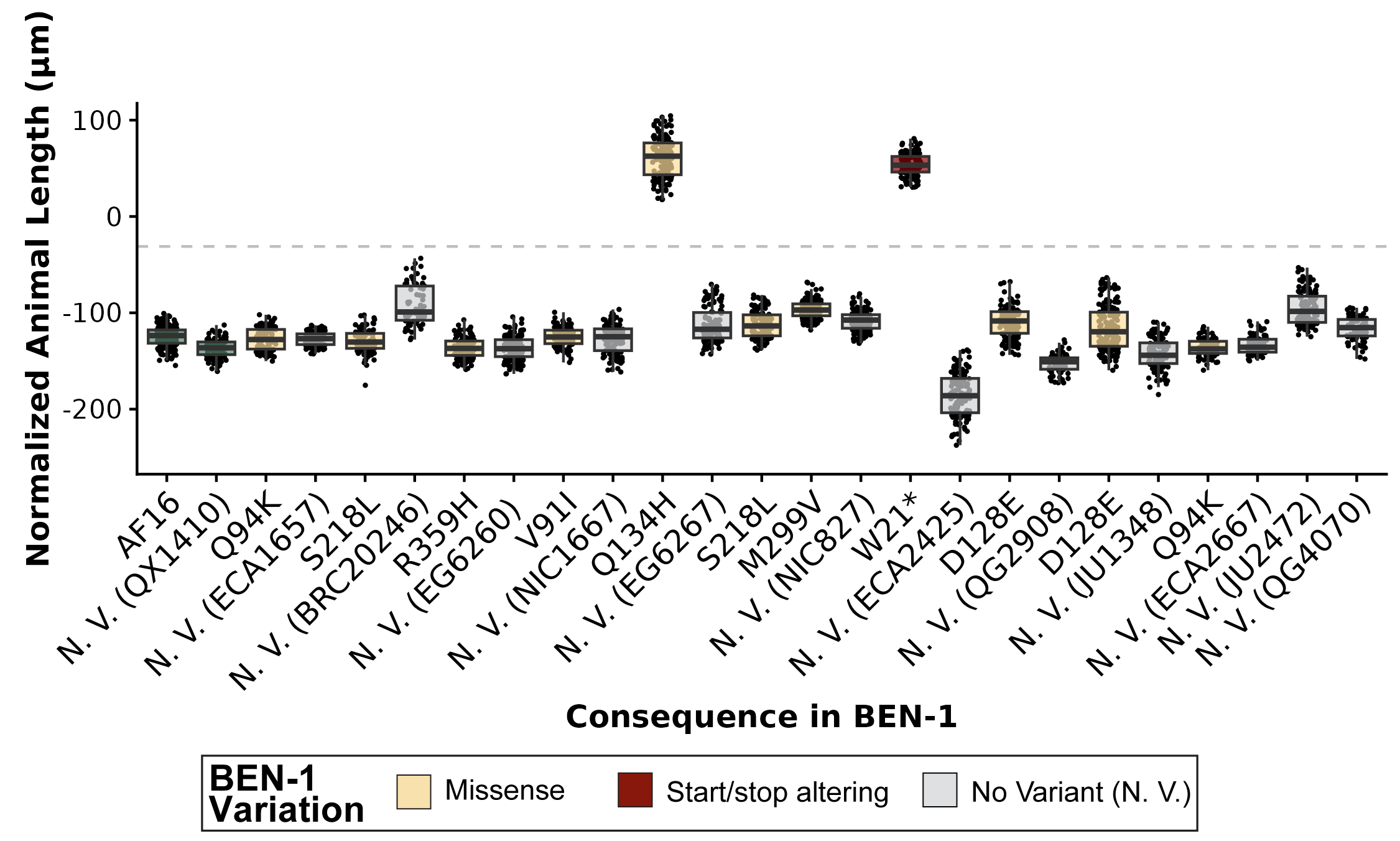

Supplement: S8 Fig — The regressed median animal length values for populations of nematodes grown in 30 μM albendazole (ABZ) are shown on the y-axis. Each point represents the normalized median animal length value of a well containing approximately five to 30 animals. Strains are sorted by their relative resistance to ABZ based on median animal length. Data are shown as Tukey box plots with the median as a solid horizontal line, and the top and bottom of the box representing the 75th and 25th quartiles, respectively. The top whisker is extended to the maximum point that is within the 1.5 interquartile range from the 75th quartile. The bottom whisker is extended to the minimum point that is within the 1.5 interquartile range from the 25th quartile. The gray dashed line marks the C. briggsae resistance threshold, defined as two standard deviations below the mean of the ben-1 deletion strain in the AF16 reference strain background. No variant (N. V.) strains (gray) paired with strains that have a high-impact variant in the ben-1 gene are shown alongside each corresponding strain with a high-impact variant in ben-1. Wild C. briggsae strains are colored by beta-tubulin variant status. (TIF) [file ppat.1014306.s008.tif]

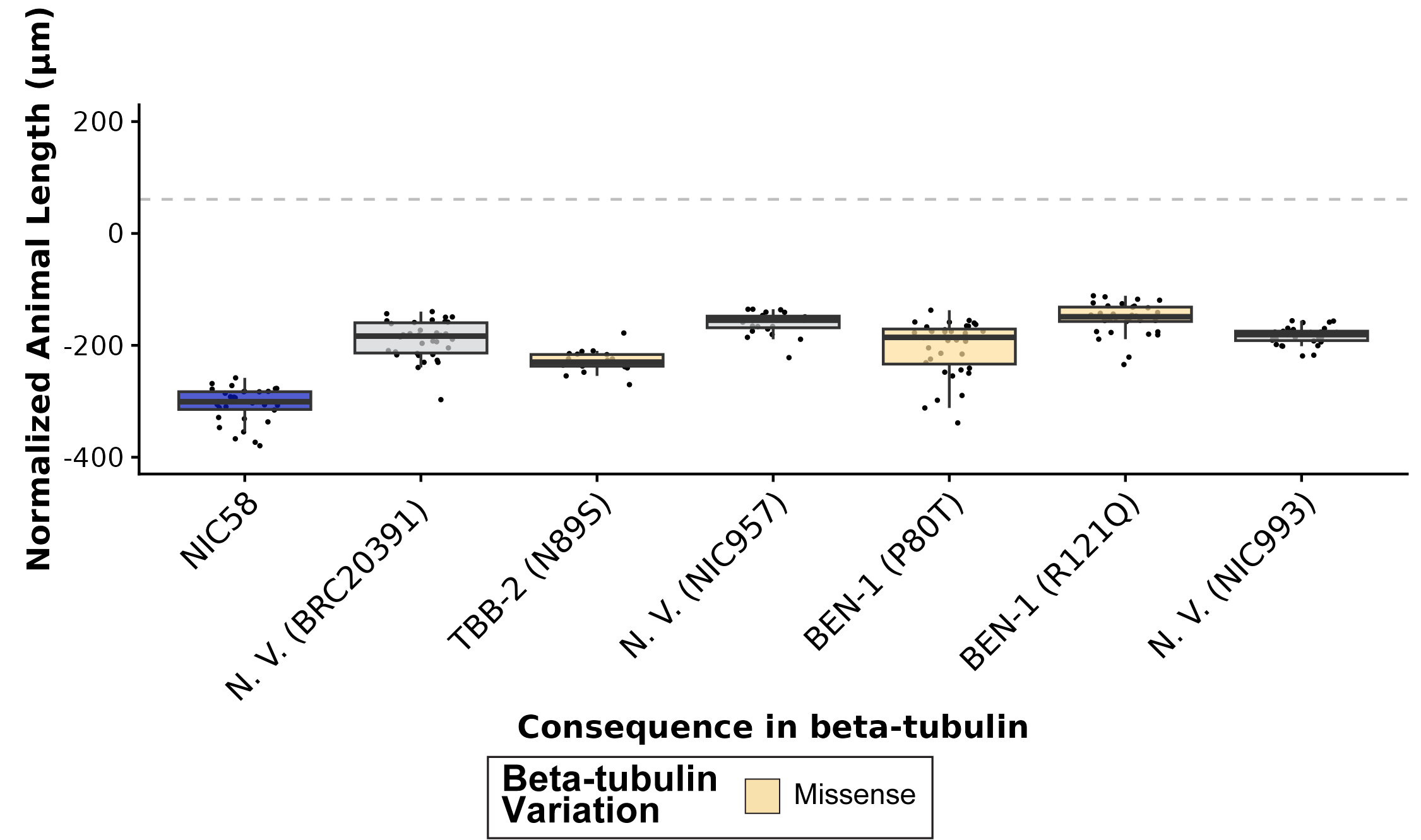

Supplement: S9 Fig — The regressed median animal length values for populations of nematodes grown in 30 μM albendazole (ABZ) are shown on the y-axis. Each point represents the normalized median animal length value of a well containing approximately five to 30 animals. Data are shown as Tukey box plots with the median as a solid horizontal line, and the top and bottom of the box representing the 75th and 25th quartiles, respectively. The top whisker is extended to the maximum point that is within the 1.5 interquartile range from the 75th quartile. The bottom whisker is extended to the minimum point that is within the 1.5 interquartile range from the 25th quartile. The gray dashed line marks the C. tropicalis resistance threshold, defined as two standard deviations below the mean of the ben-1 deletion strain in the NIC58 reference strain background. No variant (N. V.) strains (gray) paired with strains that have a high-impact variant in the tbb-2 or ben-1 genes are shown alongside each corresponding strain with a high-impact variant in tbb-2 or ben-1. Wild C. tropicalis strains are colored by beta-tubulin variant status. (TIF) [file ppat.1014306.s009.tif]

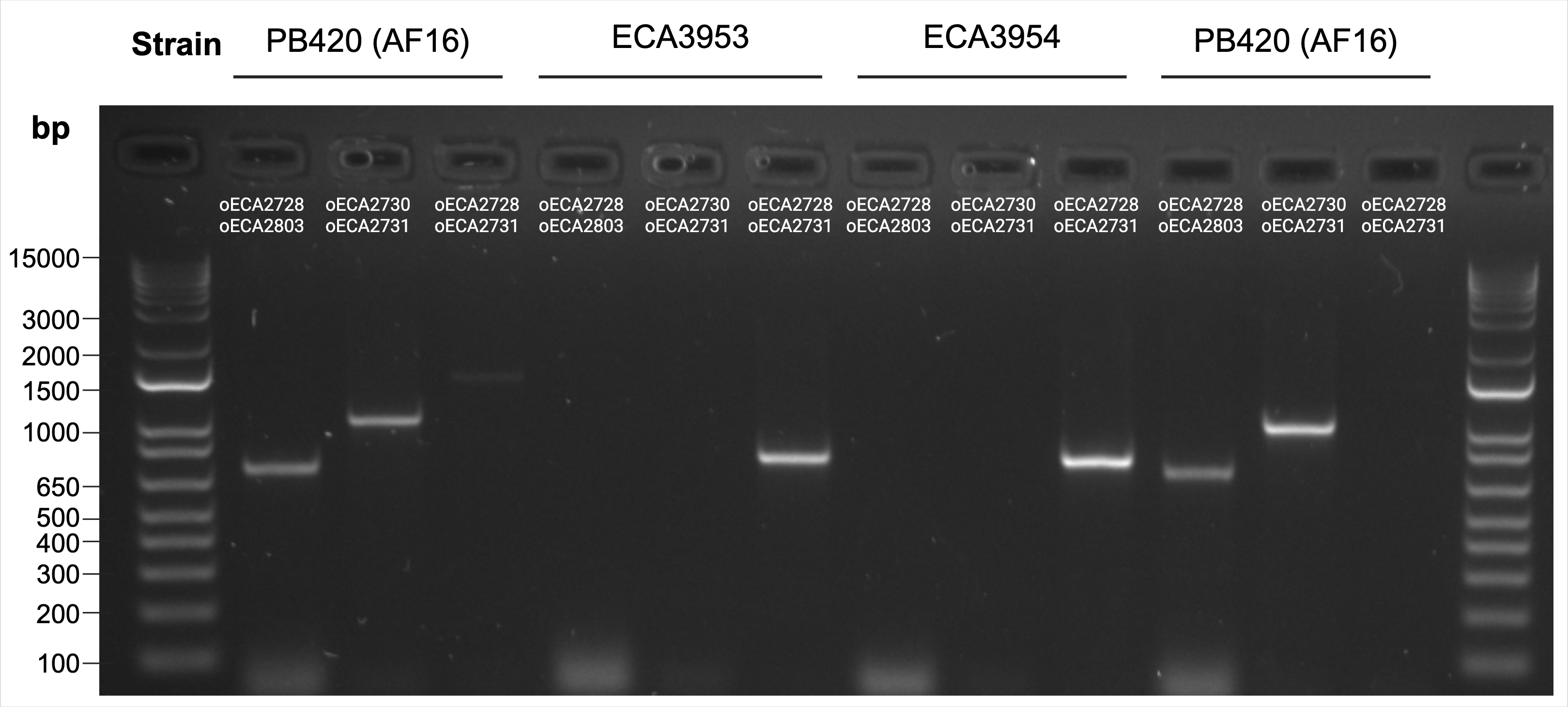

Supplement: S10 Fig — Three primer pairs were used to confirm the deletion of ben-1 in the C. briggsae reference strain, AF16. The oECA2728 (external) and oECA2803 (internal) primers flank either side of the guide region on the 5’ end. The oECA2730 (internal) and oECA2731 (external) primers flank either side of the guide region on the 3’ end. The oECA2728 and oECA2731 primers flank the outside of the ben-1 region to be deleted. The wild-type (AF16) region spans 1383 base pairs (bp), while the ben-1 deletion is reduced to 732 bp. The top of the gel is labeled by the three strains: PB420 (AF16) and the two independently edited ben-1 deletion strains in the AF16 background (ECA3953 and ECA3954). Each well of the gel is labeled by the primer pair used. The Invitrogen 1 Kb Plus DNA Ladder is shown on each side of the gel. (TIFF) [file ppat.1014306.s010.tiff]

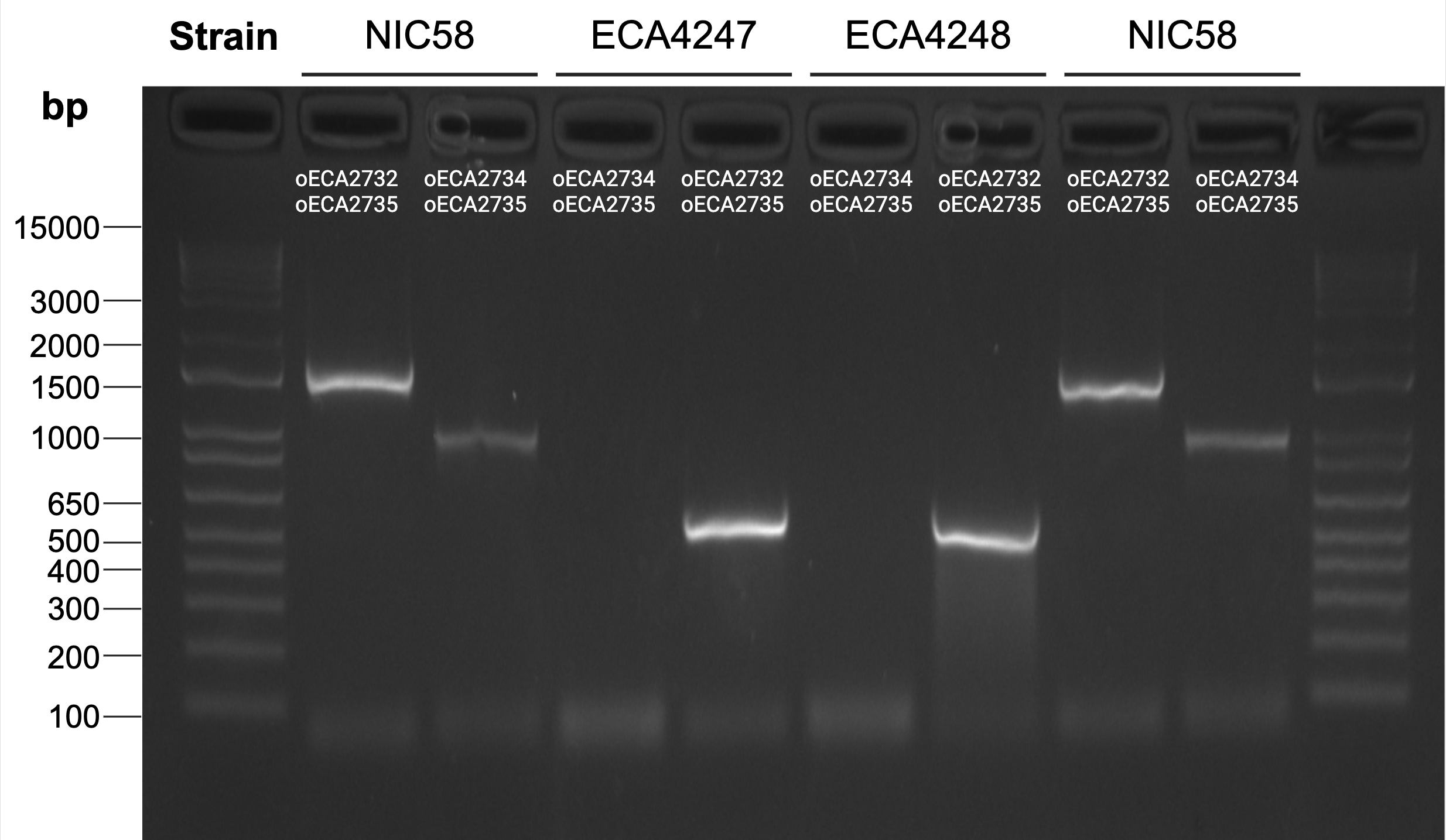

Supplement: S11 Fig — Three primer pairs were used to confirm the deletion of ben-1 in the C. tropicalis reference strain background, NIC58. The oECA2734 (internal) and oECA2735 (external) primers flank either side of the guide region on the 3’ end. The oECA2732 and oECA2735 primers flank the ben-1 region to be deleted. The wild-type (NIC58) region spans 1538 base pairs (bp), and the ben-1 deletion reduces the region to 513 bp. The top of the gel is labeled by the three strains: NIC58 and the two independently edited ben-1 deletion strains in the NIC58 background (ECA4247 and ECA4248). Each well of the gel is labeled by the primer pair used. The Invitrogen 1 Kb Plus DNA Ladder is shown on each side of the gel. (TIFF) [file ppat.1014306.s011.tiff]

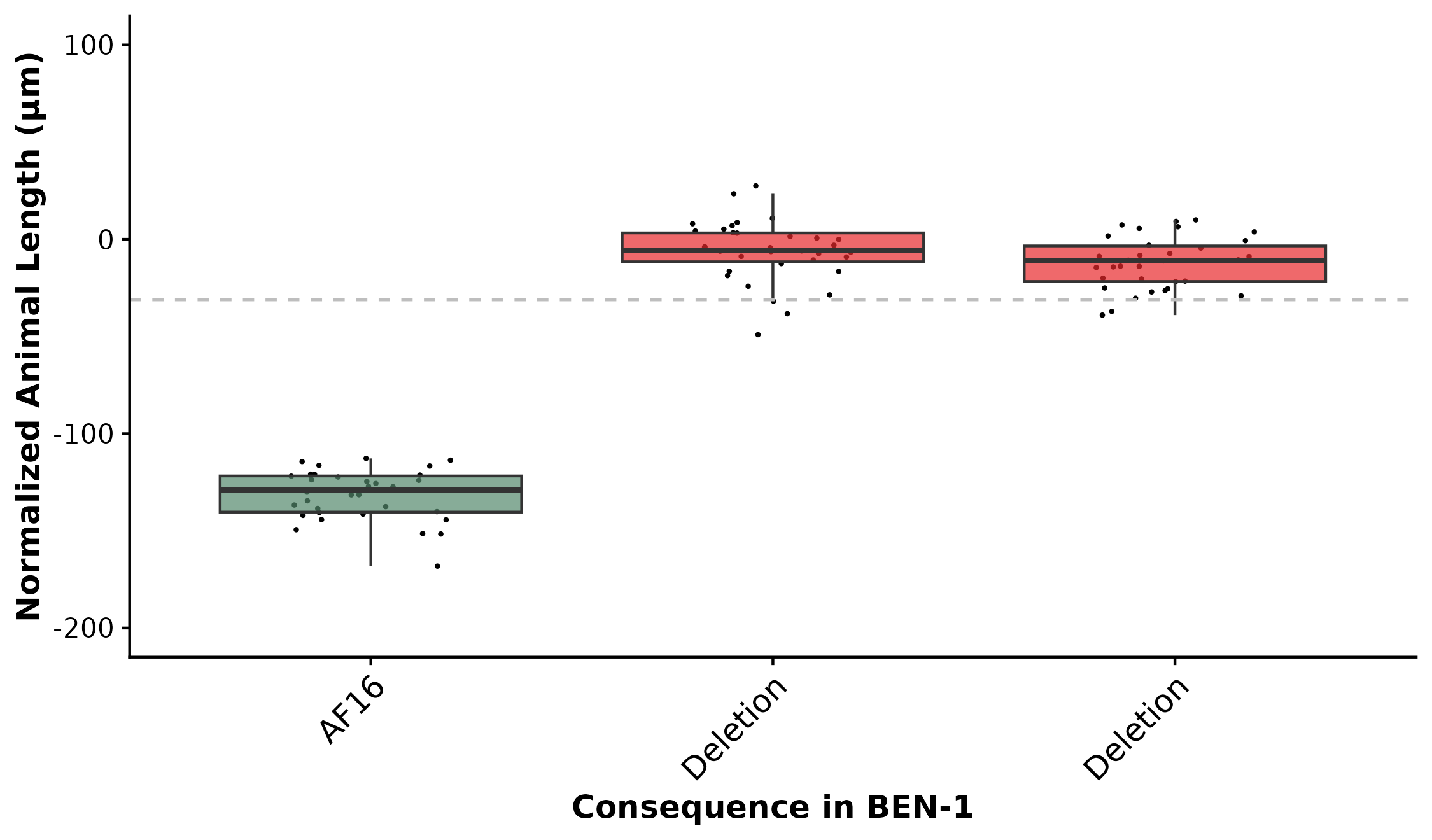

Supplement: S12 Fig — The regressed median animal length values for populations of nematodes grown in 30 μM albendazole (ABZ) are shown on the y-axis. Each point represents the normalized median animal length value of a well containing approximately five to 30 animals. Data are shown as Tukey box plots with the median as a solid horizontal line, and the top and bottom of the box representing the 75th and 25th quartiles, respectively. The top whisker is extended to the maximum point that is within the 1.5 interquartile range from the 75th quartile. The bottom whisker is extended to the minimum point that is within the 1.5 interquartile range from the 25th quartile. The gray dashed line marks the C. briggsae resistance threshold, defined as two standard deviations below the mean of the ben-1 deletion strain (ECA3953) in the AF16 reference strain background. Results are shown for the AF16 reference strain (green) and two independently edited strains with a ben-1 deletion in the AF16 background (ECA3953 and ECA3954) (red). (TIFF) [file ppat.1014306.s012.tiff]

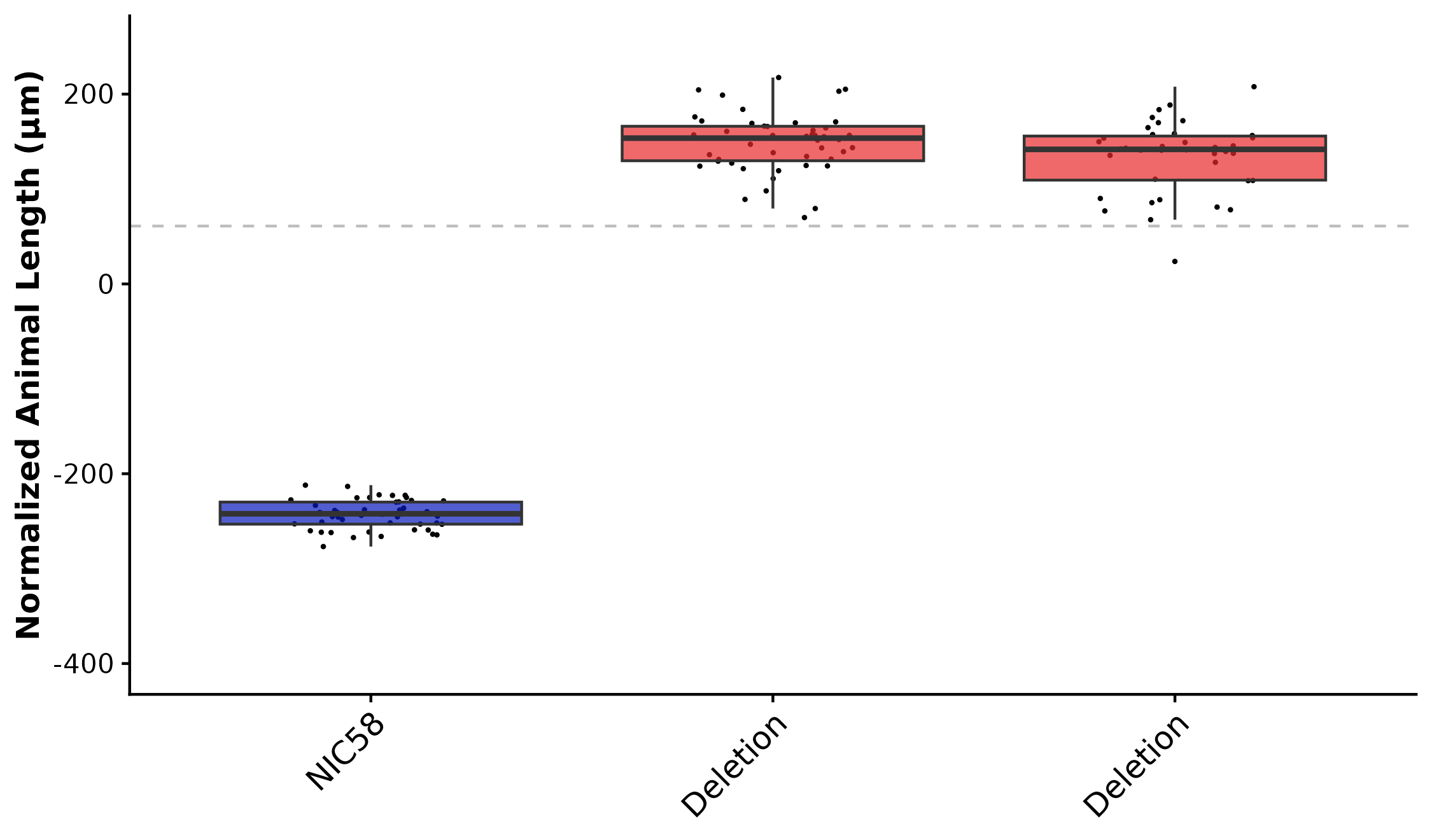

Supplement: S13 Fig — The regressed median animal length values for populations of nematodes grown in 30 μM albendazole (ABZ) are shown on the y-axis. Each point represents the normalized median animal length value of a well containing approximately five to 30 animals. Data are shown as Tukey box plots with the median as a solid horizontal line, and the top and bottom of the box representing the 75th and 25th quartiles, respectively. The top whisker is extended to the maximum point that is within the 1.5 interquartile range from the 75th quartile. The bottom whisker is extended to the minimum point that is within the 1.5 interquartile range from the 25th quartile. The gray dashed line marks the C. tropicalis resistance threshold, defined as two standard deviations below the mean of the ben-1 deletion strain (ECA24248) in the NIC58 reference strain background. Results are shown for the NIC58 reference strain (blue) and two independently edited strains with a ben-1 deletion in the NIC58 background (ECA4247 and ECA4248) (red). (TIFF) [file ppat.1014306.s013.tiff]

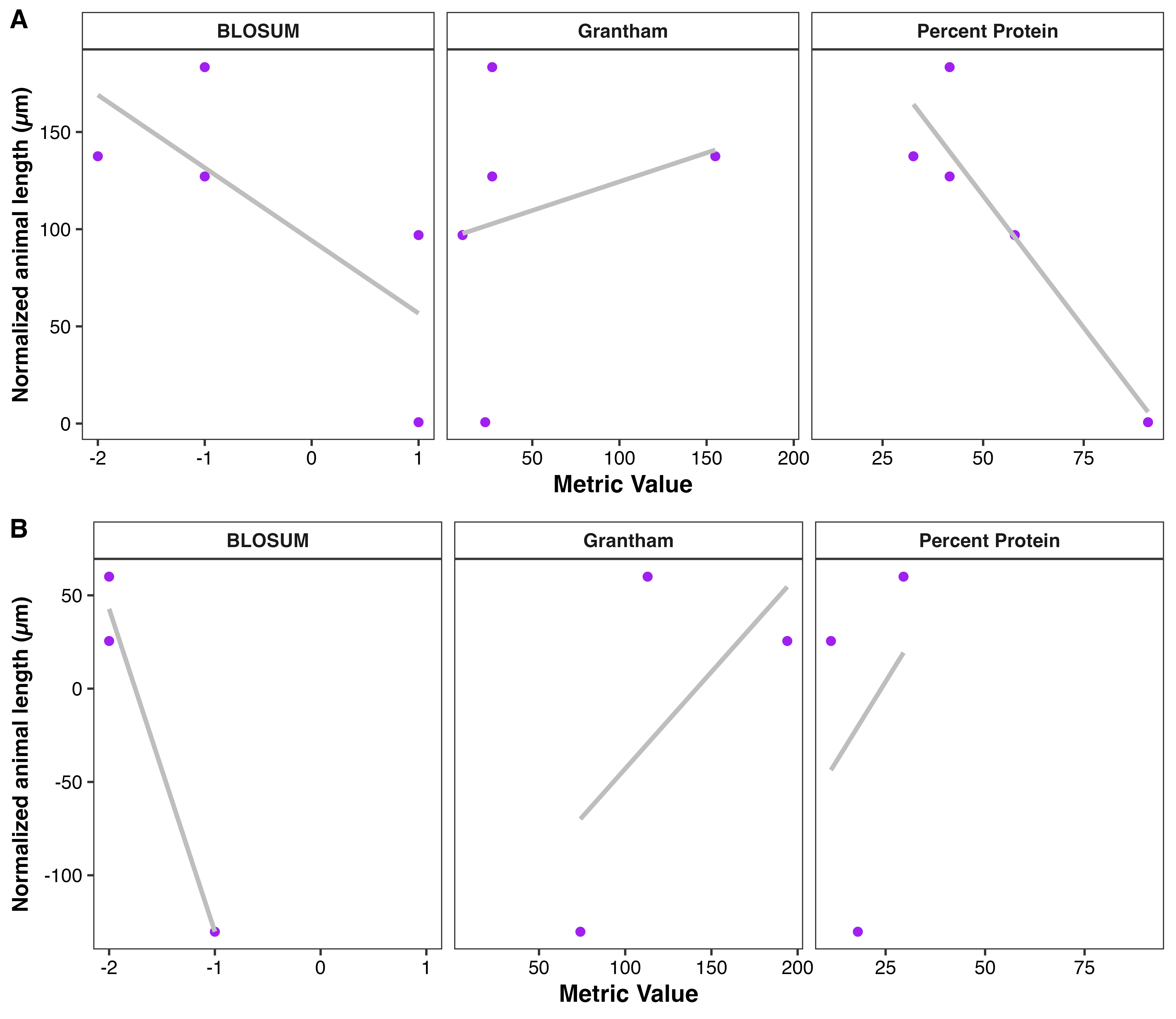

Supplement: S14 Fig — Scatterplots show the relationship between normalized median animal length (y-axis) and three amino acid substitution scoring metrics (x-axis): BLOSUM62 (R2 = 0.97, p-value = 0.11), Grantham (R2 = 0.39, p-value = 0.57), and percent protein (R2 = 0.1, p-value = 0.8). Each point represents a C. elegans strain with a missense substitution in BEN-1. Gray lines indicate the linear regression fit for these models. (A) Strains phenotyped for ABZ response in previous assays are plotted [14, 36]. (B) Strains phenotyped in the assays performed for this study are plotted. (TIFF) [file ppat.1014306.s014.tiff]

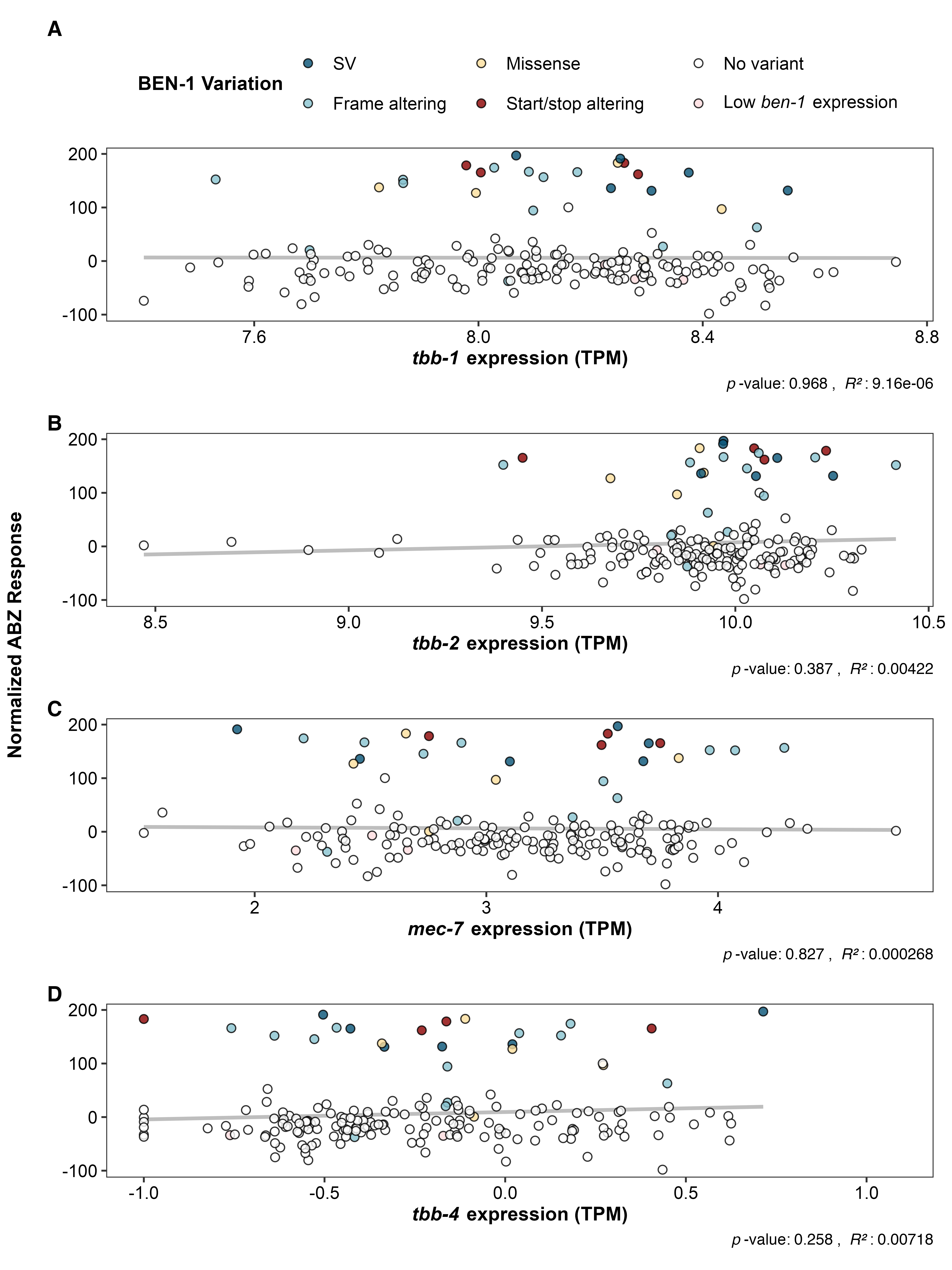

Supplement: S15 Fig — Scatterplot of the relationship between (A) tbb-1, (B) tbb-2, (C) mec-7, and (D) tbb-4 expression levels and normalized albendazole (ABZ) response across C. elegans wild strains. Each point represents a strain phenotyped for ABZ response in previous publications (Hahnel et al., 2018; Shaver et al., 2024) with tbb-1, tbb-2, mec-7, and tbb-4 expression data [37]. The tbb-1, tbb-2, mec-7, and tbb-4 expression levels measured in transcripts per million (TPM) are displayed on the x-axis. The normalized ABZ response values adjusted for assay-specific effects are displayed on the y-axis. The gray line represents the linear regression fit between beta-tubulin gene expression and normalized response, with the linear model’s coefficient of determination (R²). Data points are colored based on the predicted functional consequence of their ben-1 alleles (i.e., large structural variant (SV), frameshift, missense substitution, disrupted start/stop sequence, no high-impact variant, or low ben-1 expression). (TIFF) [file ppat.1014306.s015.tiff]

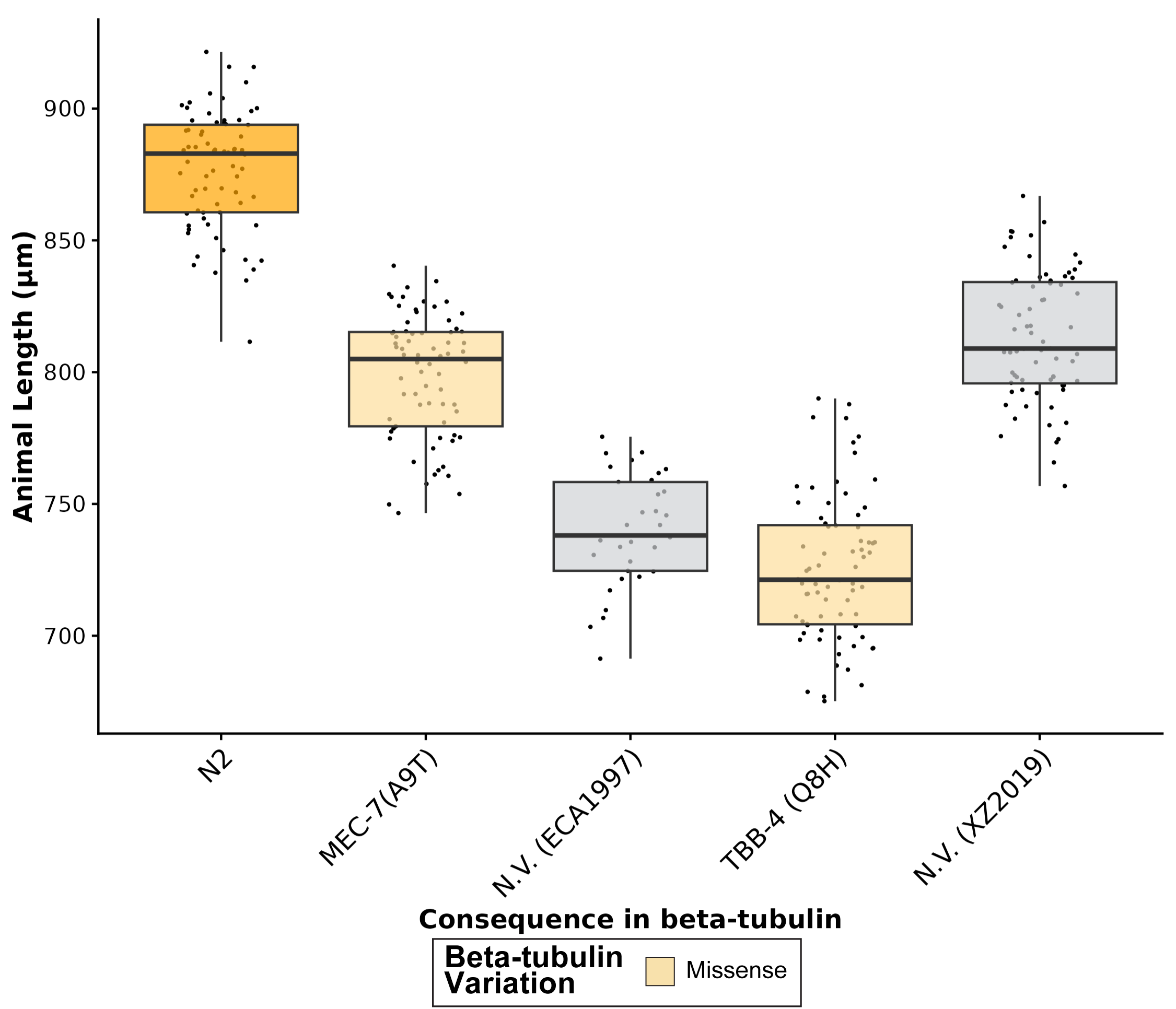

Supplement: S16 Fig — Median animal length values from populations of nematodes grown in DMSO are shown on the y-axis. Each point represents the median animal length from a well containing approximately five to 30 animals. Data are shown as Tukey box plots with the median as a solid horizontal line, the top and bottom of the box representing the 75th and 25th quartiles, respectively. The top whisker is extended to the maximum point that is within a 1.5 interquartile range from the 75th quartile. The bottom whisker is extended to the minimum point that is within the 1.5 interquartile range from the 25th quartile. No variant (N. V.) strains (gray) paired with strains that have a high-impact variant in a beta-tubulin gene are shown alongside each corresponding strain with a high-impact variant in a beta-tubulin gene. Wild C. elegans strains are colored by beta-tubulin variant status. (TIF) [file ppat.1014306.s016.tif]

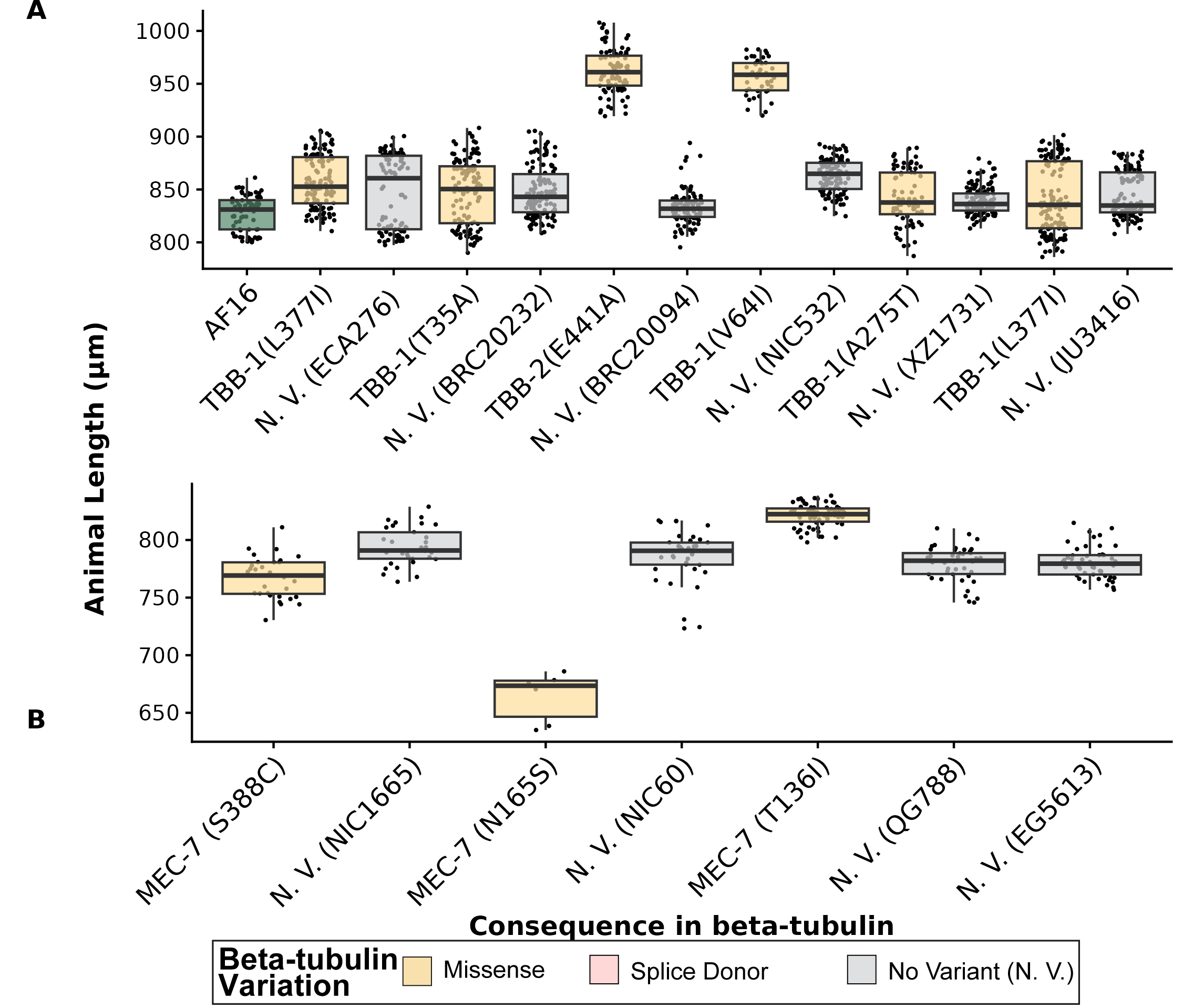

Supplement: S17 Fig — Median animal length values from populations of nematodes grown in DMSO are shown on the y-axis. Each point represents the median animal length from a well containing approximately five to 30 animals. Data are shown as Tukey box plots with the median as a solid horizontal line, the top and bottom of the box representing the 75th and 25th quartiles, respectively. The top whisker is extended to the maximum point that is within a 1.5 interquartile range from the 75th quartile. The bottom whisker is extended to the minimum point that is within the 1.5 interquartile range from the 25th quartile. No variant (N. V.) strains (gray) paired with strains that have a high-impact variant in a beta-tubulin gene are shown alongside each corresponding strain with a high-impact variant in a beta-tubulin gene. Wild C. briggsae strains are colored by beta-tubulin variant status. (TIF) [file ppat.1014306.s017.tif]

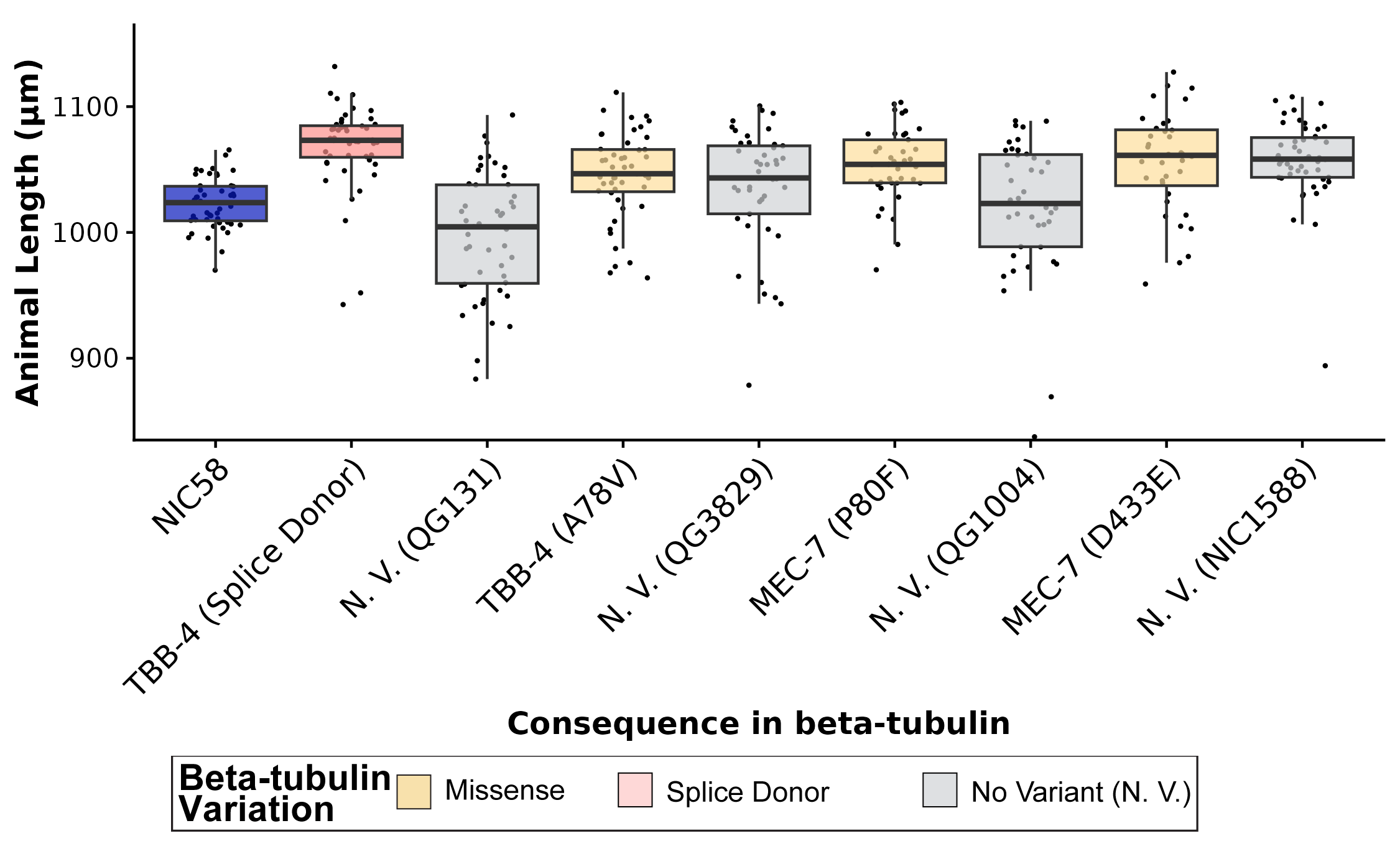

Supplement: S18 Fig — Median animal length values from populations of nematodes grown in DMSO are shown on the y-axis. Each point represents the median animal length from a well containing approximately five to 30 animals. Data are shown as Tukey box plots with the median as a solid horizontal line, the top and bottom of the box representing the 75th and 25th quartiles, respectively. The top whisker is extended to the maximum point that is within a 1.5 interquartile range from the 75th quartile. The bottom whisker is extended to the minimum point that is within the 1.5 interquartile range from the 25th quartile. No variant (N. V.) strains (gray) paired with strains that have a high-impact variant in a beta-tubulin gene are shown alongside each corresponding strain with a high-impact variant in a beta-tubulin gene. Wild C. tropicalis strains are colored by beta-tubulin variant status. (TIF) [file ppat.1014306.s018.tif]

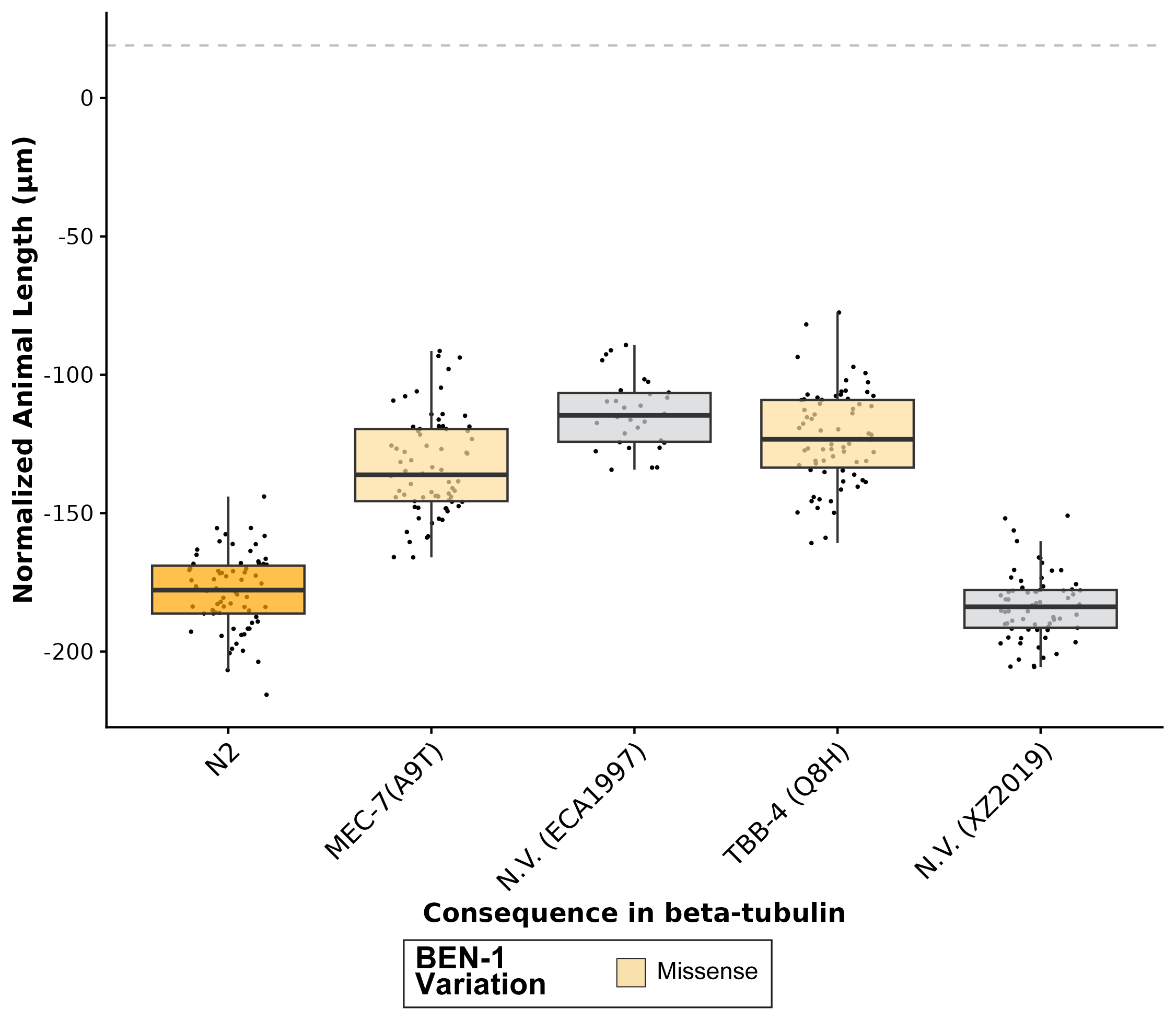

Supplement: S19 Fig — The regressed median animal length values for populations of nematodes grown in 30 μM albendazole (ABZ) are shown on the y-axis. Each point represents the normalized median animal length value of a well containing approximately five to 30 animals. Data are shown as Tukey box plots with the median as a solid horizontal line, and the top and bottom of the box representing the 75th and 25th quartiles, respectively. The top whisker is extended to the maximum point that is within the 1.5 interquartile range from the 75th quartile. The gray dashed line marks the C. elegans resistance threshold, defined as two standard deviations below the mean of the ben-1 deletion strain in the N2 reference strain background. The bottom whisker is extended to the minimum point that is within the 1.5 interquartile range from the 25th quartile. No variant (N. V.) strains (gray) paired with strains that have a high-impact variant in a beta-tubulin gene are shown alongside each corresponding strain with a high-impact variant in a beta-tubulin gene. Wild C. elegans strains are colored by beta-tubulin variant status. (TIF) [file ppat.1014306.s019.tif]

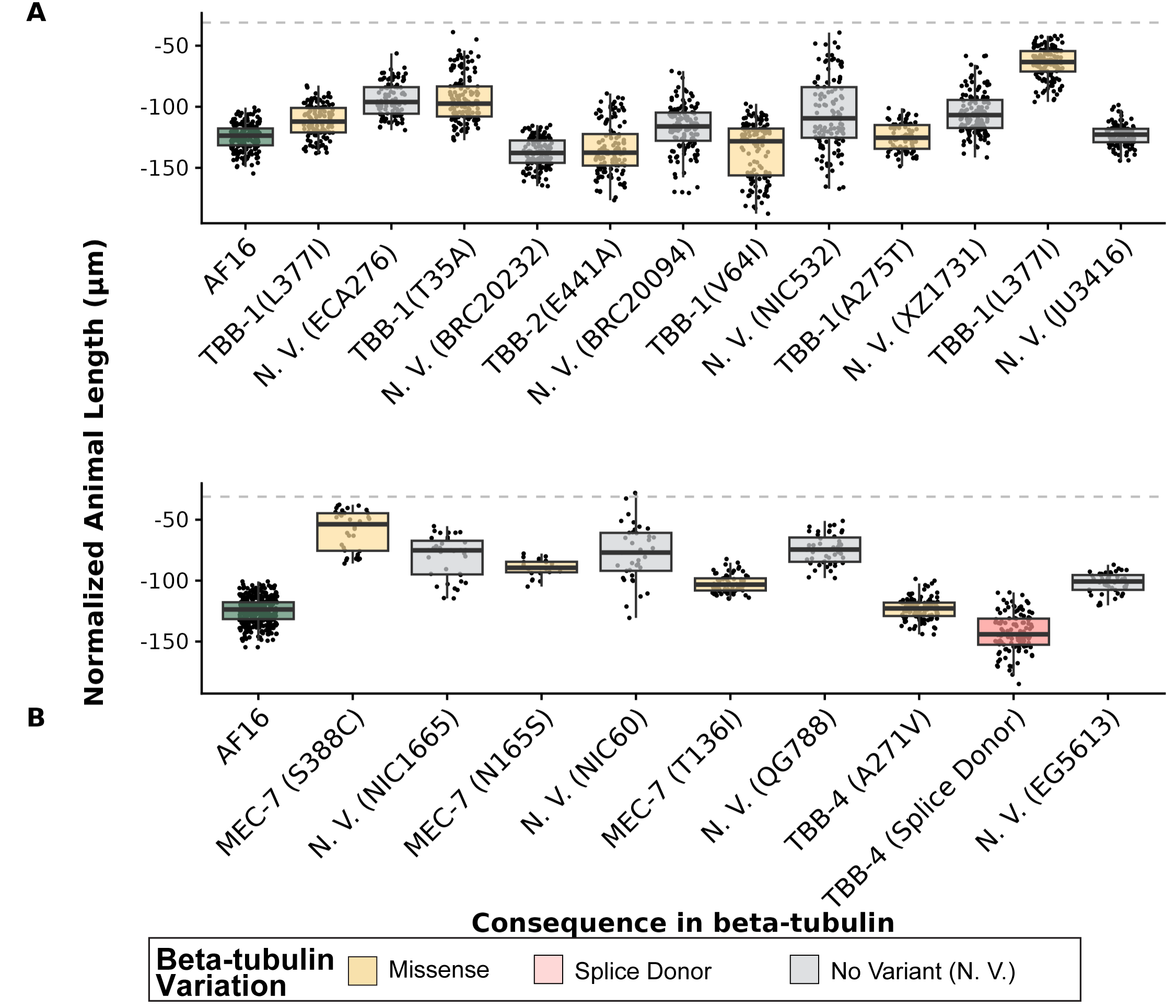

Supplement: S20 Fig — The regressed median animal length values for populations of nematodes grown in 30 μM albendazole (ABZ) are shown on the y-axis. Each point represents the normalized median animal length value of a well containing approximately five to 30 animals. Data are shown as Tukey box plots with the median as a solid horizontal line, and the top and bottom of the box representing the 75th and 25th quartiles, respectively. The top whisker is extended to the maximum point that is within the 1.5 interquartile range from the 75th quartile. The bottom whisker is extended to the minimum point that is within the 1.5 interquartile range from the 25th quartile. The gray dashed line marks the C. briggsae resistance threshold, defined as two standard deviations below the mean of the ben-1 deletion strain in the AF16 reference strain background. Results for the AF16 reference strain and all C. briggsae wild strains with unique high-impact variants in (A) TBB-1 and TBB-2 and (B) TBB-4 and MEC-7 are shown. No variant (N. V.) strains (gray) paired with strains that have a high-impact variant in a beta-tubulin gene are shown alongside each corresponding strain with a high-impact variant in a beta-tubulin gene. Wild C. tropicalis strains are colored by beta-tubulin variant status. (TIF) [file ppat.1014306.s020.tif]

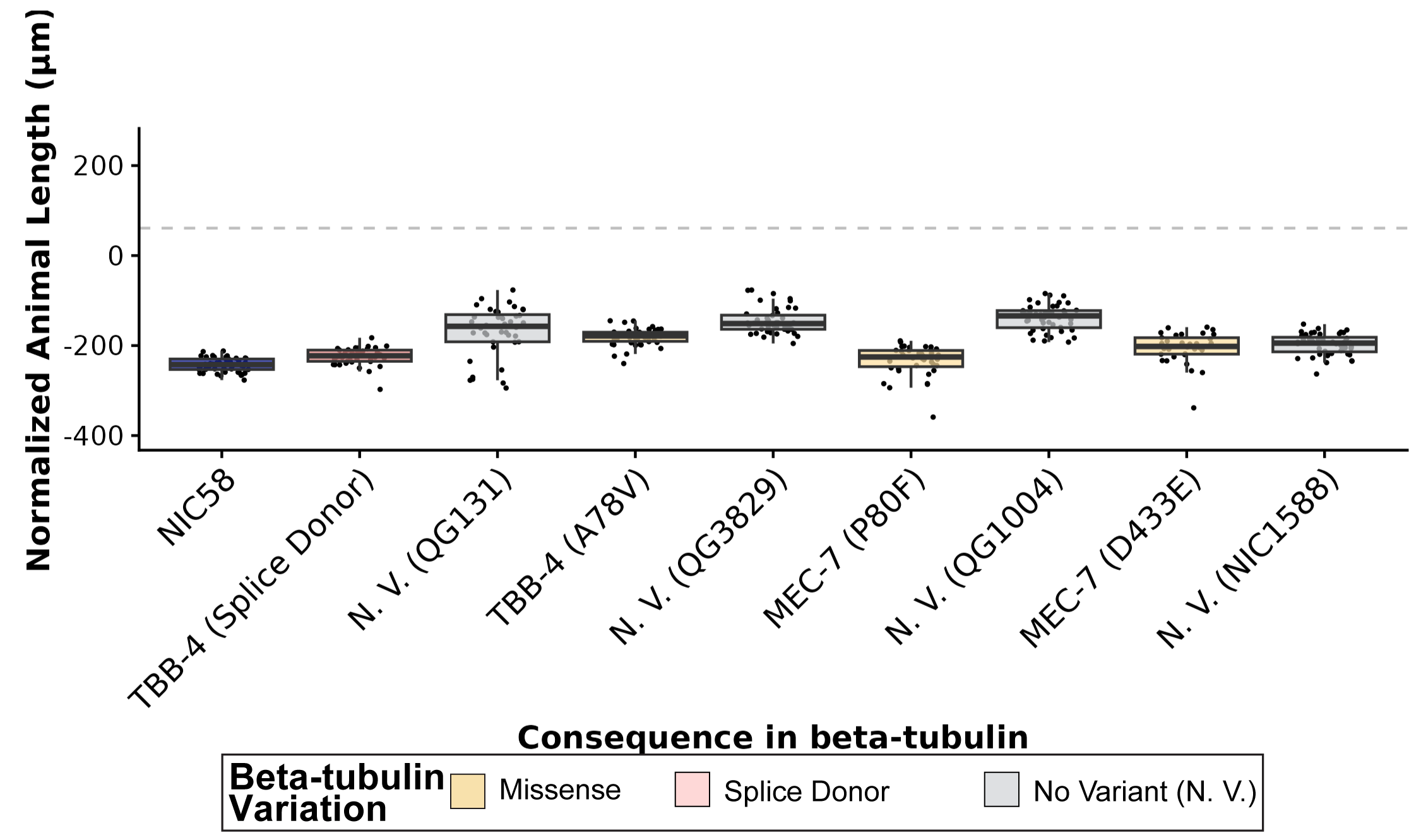

Supplement: S21 Fig — The regressed median animal length values for populations of nematodes grown in 30 μM albendazole (ABZ) are shown on the y-axis. Each point represents the normalized median animal length value of a well containing approximately five to 30 animals. Data are shown as Tukey box plots with the median as a solid horizontal line, and the top and bottom of the box representing the 75th and 25th quartiles, respectively. The top whisker is extended to the maximum point that is within the 1.5 interquartile range from the 75th quartile. The bottom whisker is extended to the minimum point that is within the 1.5 interquartile range from the 25th quartile. The gray dashed line marks the C. tropicalis resistance threshold, defined as two standard deviations below the mean of the ben-1 deletion strain in the NIC58 reference strain background. No variant (N. V.) strains (gray) paired with strains that have a high-impact variant in a beta-tubulin gene are shown alongside each corresponding strain with a high-impact variant in a beta-tubulin gene. Wild C. tropicalis strains are colored by beta-tubulin variant status. (TIF) [file ppat.1014306.s021.tif]

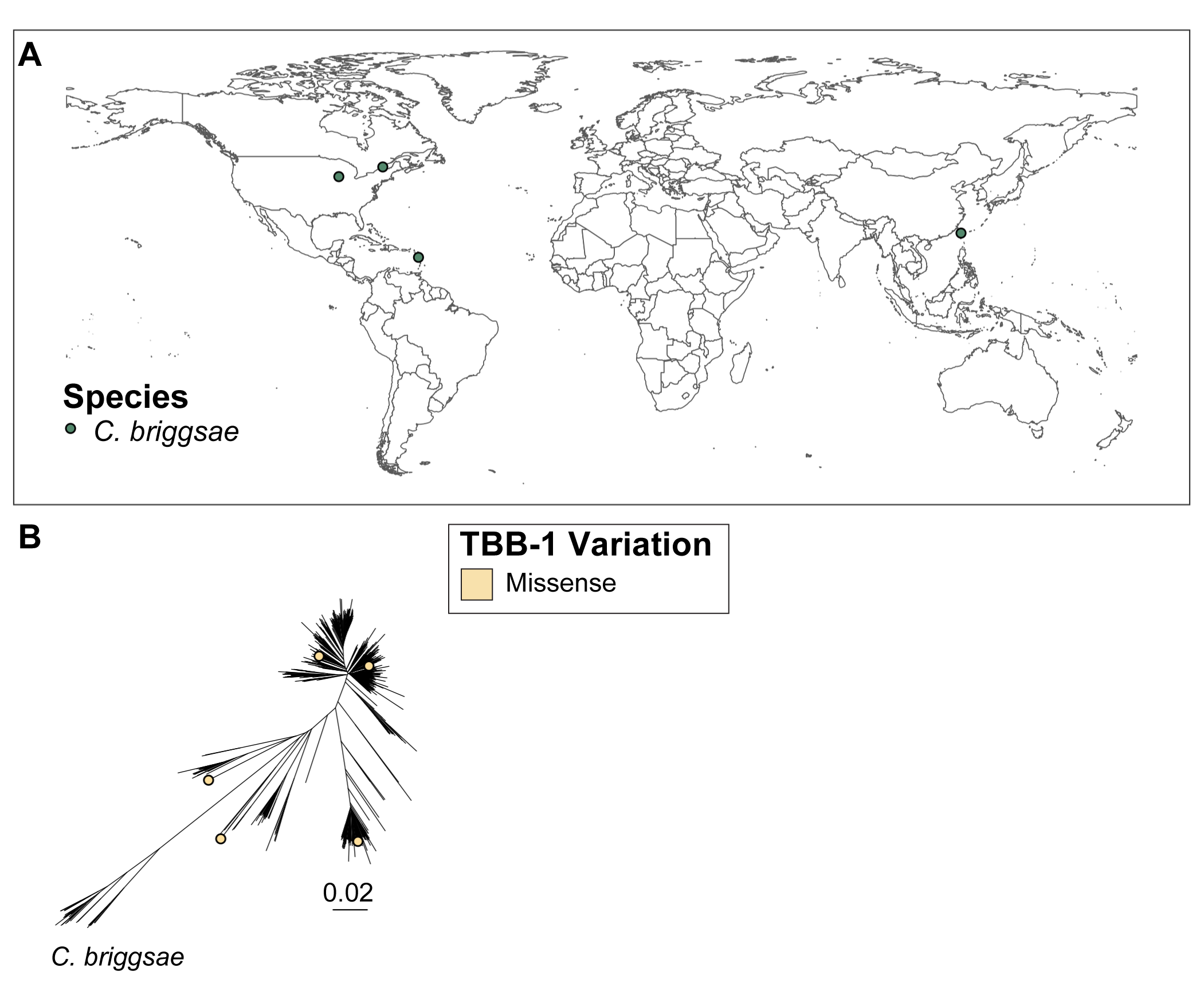

Supplement: S22 Fig — Each point represents an isotype reference strain with a predicted high-impact variant in tbb-1. (A) Each point corresponds to the sampling location of the strain. (B) Each point corresponds to the location of the strain in a genome-wide phylogeny of 641 C. briggsae isotype reference strains. One isotype, XZ1213 has a high-impact tbb-1 variant, but sampling coordinates were not recorded. The base layer of the map was obtained from the Natural Earth world countries shape file accessed via the R package rnaturalearth and function ne_countries(). The scale was set to return a medium-scale base map with the scale = medium parameter. The direct link to the base layer of the map can be accessed here: https://www.naturalearthdata.com/. (TIF) [file ppat.1014306.s022.tif]

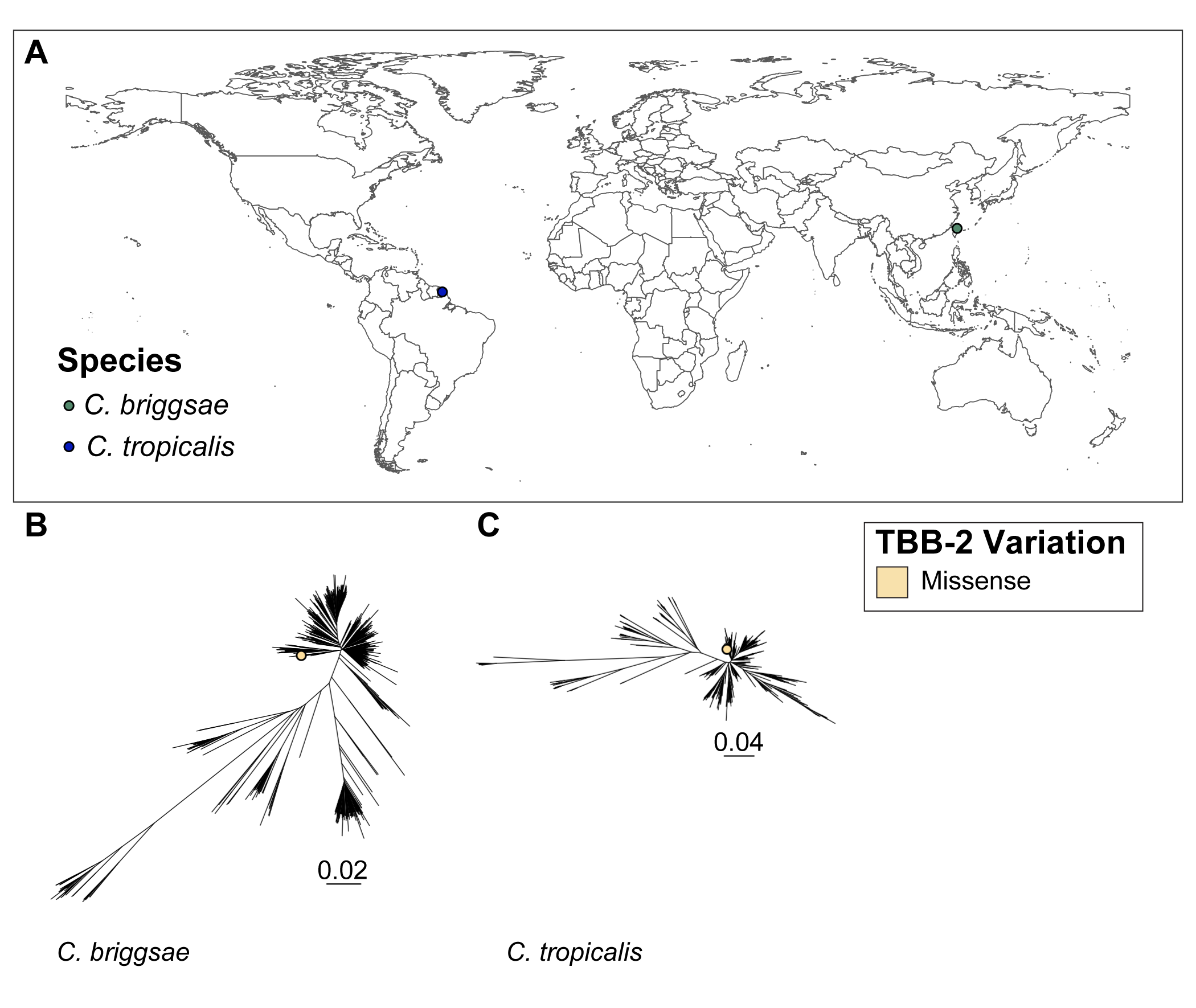

Supplement: S23 Fig — Each point represents an isotype reference strain with a predicted high-impact variant in tbb-2. (A) Each point corresponds to the sampling location of the strain. Each point corresponds to the location of the strain in a genome-wide phylogeny of (B) 641 C. briggsae and (C) 518 C. tropicalis isotype reference strains. The base layer of the map was obtained from the Natural Earth world countries shape file accessed via the R package rnaturalearth and function ne_countries(). The scale was set to return a medium-scale base map with the scale = medium parameter. The direct link to the base layer of the map can be accessed here: https://www.naturalearthdata.com/. (TIF) [file ppat.1014306.s023.tif]

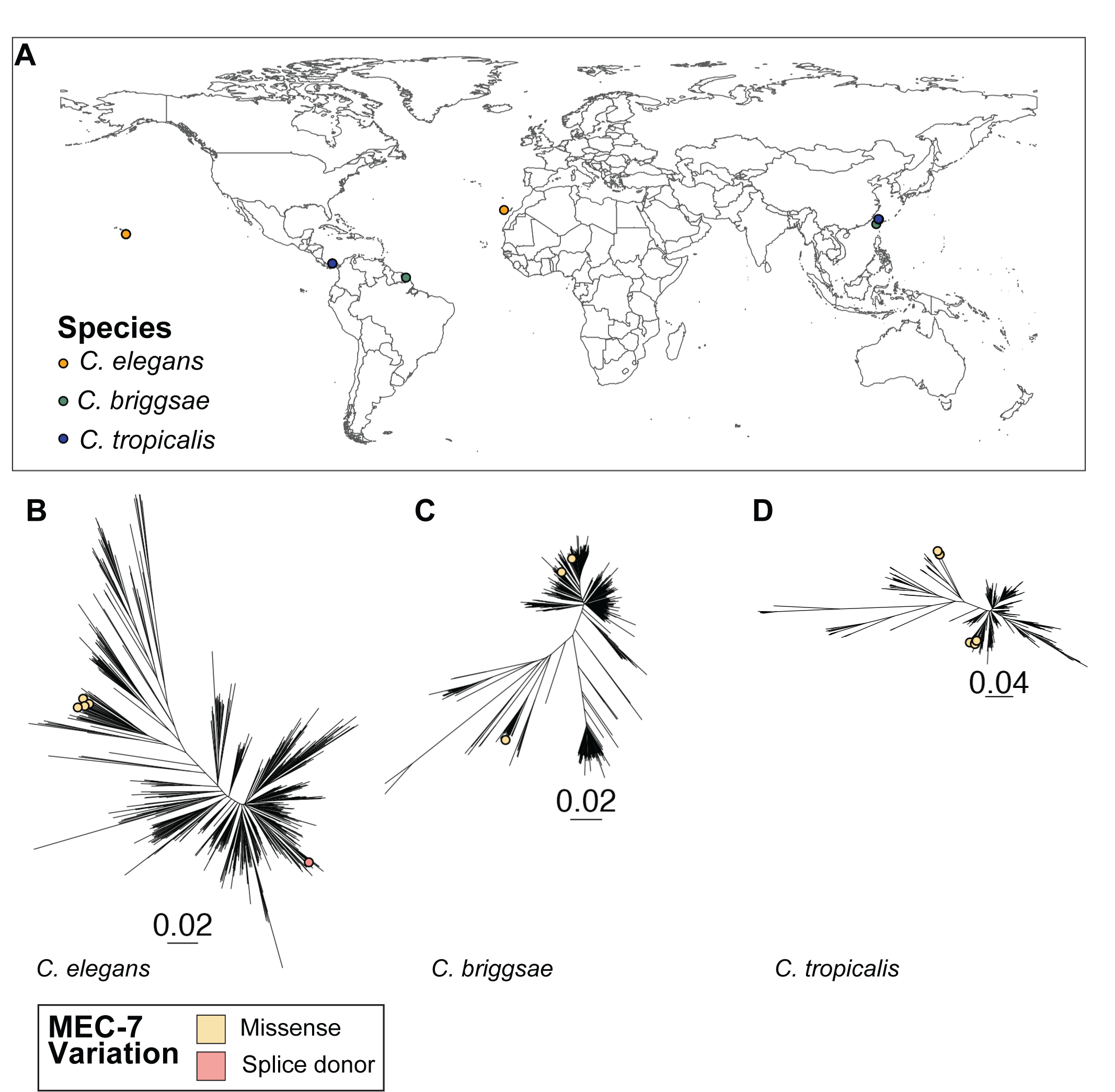

Supplement: S24 Fig — Each point represents an isotype reference strain with a predicted high-impact variant in mec-7. (A) Each point corresponds to the sampling location of an individual C. elegans, C. briggsae, or C. tropicalis strain. A genome-wide phylogeny of (B) 611 C. elegans, (C) 641 C. briggsae, and (D) 518 C. tropicalis isotype reference strains, where each point denotes an isotype reference strain with a predicted high-impact consequence in mec-7. The base layer of the map was obtained from the Natural Earth world countries shape file accessed via the R package rnaturalearth and function ne_countries(). The scale was set to return a medium-scale base map with the scale = medium parameter. The direct link to the base layer of the map can be accessed here: https://www.naturalearthdata.com/. (TIF) [file ppat.1014306.s024.tif]

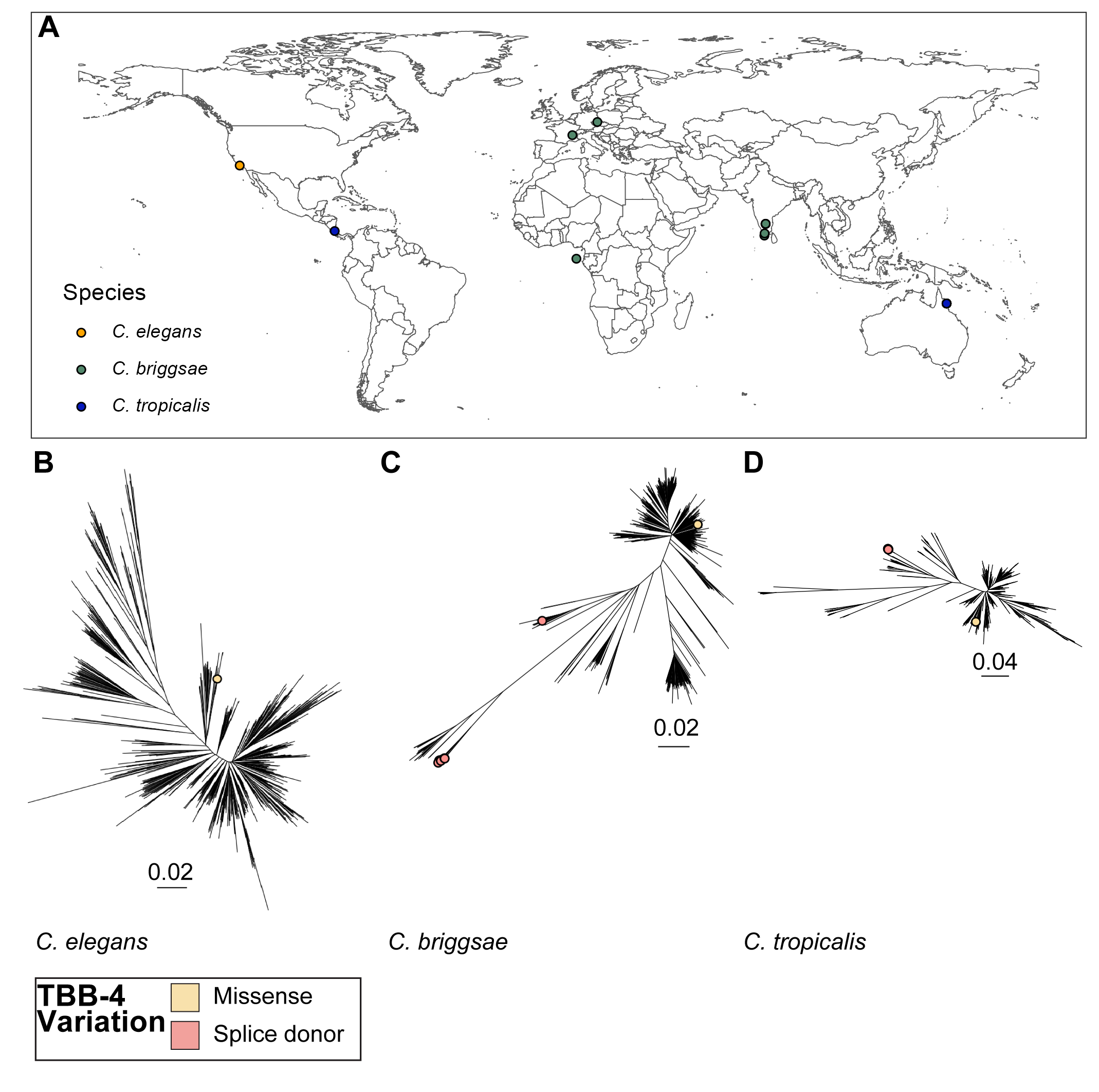

Supplement: S25 Fig — Each point represents an isotype reference strain with a predicted high-impact variant in tbb-4. (A) Each point corresponds to the sampling location of an individual C. elegans, C. briggsae, or C. tropicalis strain with a predicted high-impact consequence in the gene tbb-4. Each point corresponds to the location of the strain in a genome-wide phylogeny of (B) 611 C. elegans, (C) 641 C. briggsae, and (D) 518 C. tropicalis isotype reference strains. The base layer of the map was obtained from the Natural Earth world countries shape file accessed via the R package rnaturalearth and function ne_countries(). The scale was set to return a medium-scale base map with the scale = medium parameter. The direct link to the base layer of the map can be accessed here: https://www.naturalearthdata.com/ (TIF) [file ppat.1014306.s025.tif]

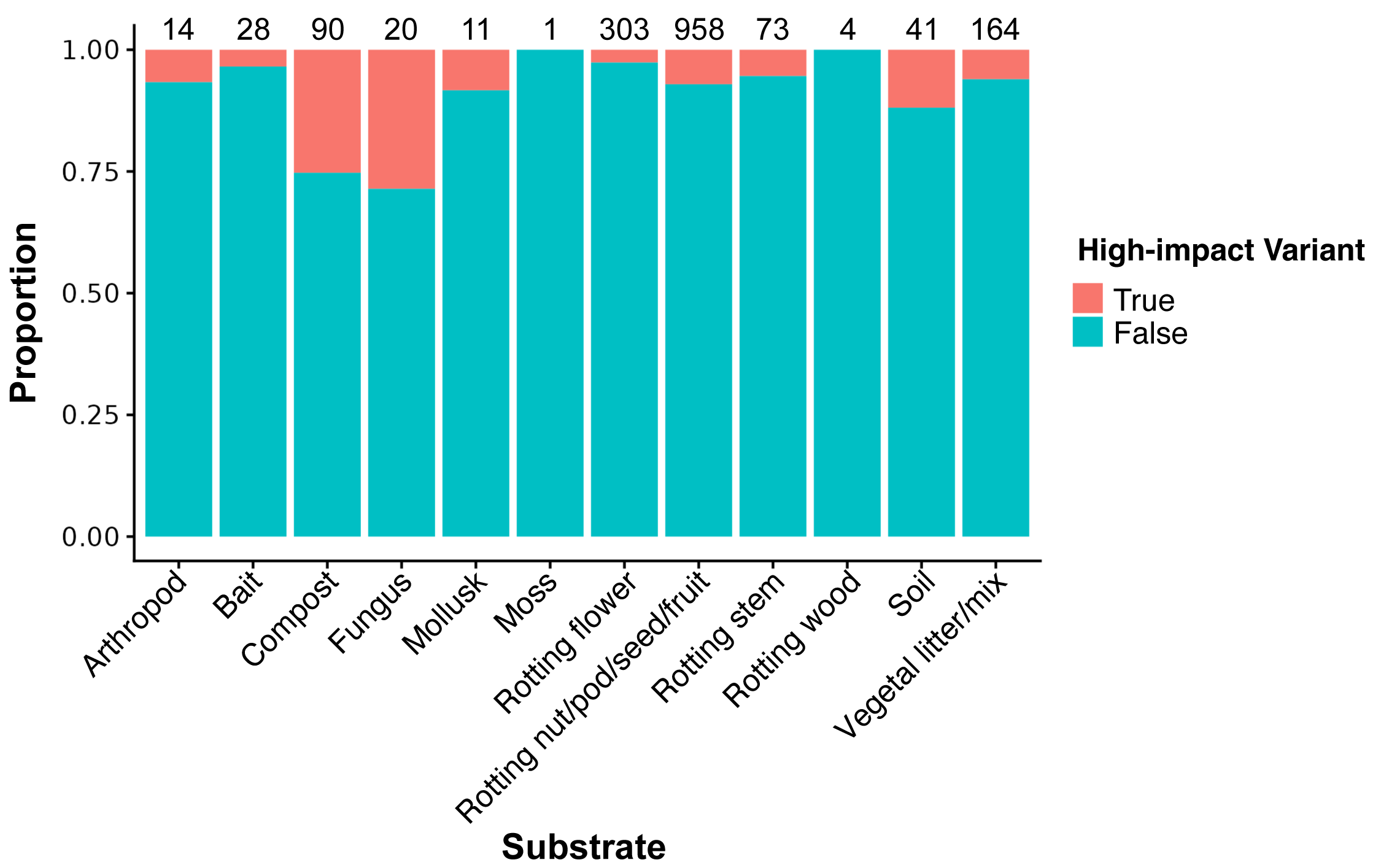

Supplement: S26 Fig — The proportion of strains (y-axis) found in a given substrate (x-axis) are displayed. Strains with a high-impact variant in a beta-tubulin gene are colored salmon. Strains with no variants in a beta-tubulin gene are colored teal. The total number of strains isolated from a given substrate is displayed above each column. Moss and rotting wood were not included in the substrate enrichment analysis due to the small sample size. No significant relationship between beta-tubulin gene variant status and substrate were identified (Fisher’s Exact Test, p = 1). (TIF) [file ppat.1014306.s026.tif]

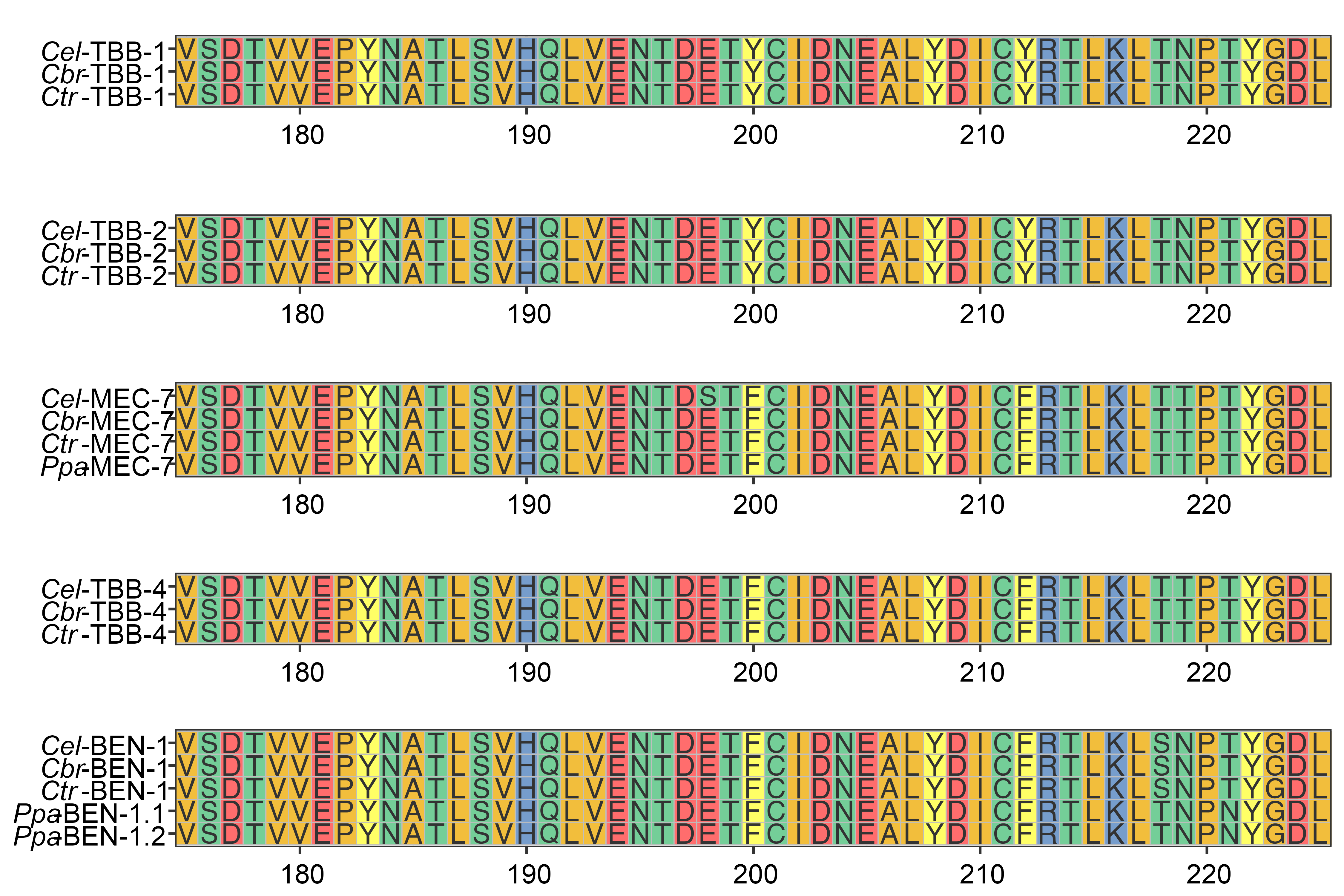

Supplement: S27 Fig — Amino acid sequences of beta-tubulin isoforms TBB-1, TBB-2, MEC-7, TBB-4, and BEN-1 from C. elegans (Cel-), C. briggsae (Cbr-), C. tropicalis (Ctr-), and P. pacificus (Ppa-) are aligned with MAFFT, and the alignment is displayed from amino acid residue 175 to residue 225. The region displayed is hypothesized to bind benzimidazoles. Residues are colored by side-chain chemical properties with the default ggmsa color scheme. (TIFF) [file ppat.1014306.s027.tiff]
